# Environmentally sustainable epoxy nanocomposite coating reinforced with chitosan derived nitrogen doped graphene for enhanced corrosion resistance and mechanical performance

**DOI:** 10.1038/s41598-025-11204-6

**Published:** 2025-08-20

**Authors:** Marwa Adel, Dalia S. Fathy, Osama Abo El-eneen

**Affiliations:** 1 Fabrication Technology Department, Advanced Technology and New Materials Research Institute, City of Scientific Research and Technological Applications (SRTA City), New Borg El-Arab, Alexandria, 21934 Egypt; 2 Petroleum Applications Department, Egyptian Petroleum Research Institute (EPRI), Nasr City, Cairo, 11727 Egypt; 3 Petrochemicals Department, Polymer Laboratory, Egyptian Petroleum Research Institute (EPRI), Nasr City, Egypt

**Keywords:** Graphene, Solvothermal, Anticorrosion coating, Toughness, Mechanical performance, Environmental sciences, Chemistry, Materials science

## Abstract

The current study evaluates the anticorrosive performance of Epoxy coating augmented with a distinctive, environmentally safe, nitrogen-doped graphene. A simple, green, and single-step cost-effective route for synthesis graphene nanosheets doped with nitrogen (Gr) has been developed using direct-solvothermal treatment of chitosan, under gentle conditions. XRD, FTIR, SEM, XPS and Raman spectroscopy were employed to characterize the microstructure characteristics of N-doped graphene. Well separated flatten morphology with folding characteristics and extremely extended considerable average lateral dimensions further than 1.27 mm was detected. Graphene was successfully integrated into epoxy coatings on carbon steel substrates with different tiny graphene concentrations < 0.04 wt%. The coatings’ corrosion resistance capabilities and accelerated durability tests were studied, including salt spray corrosion, bending, impact, adhesion, abrasion properties, wear resistance, and hardness. Comprehensive mechanical performance was investigated. Understanding the microstructural characteristics of Gr, the interface character of Gr/Epoxy coating composites with different Nanofiller loading and how these affect both the mechanism of the enhancement of corrosion protection process and their tensile behavior, were a focal point of interest. Remarkably, the coating with just 0.02 wt% Gr exhibited a 70% reduction in wear index compared to neat epoxy, along with significant enhancements in mechanical toughness showing a 573% increase in ultimate toughness and a 993% increase in toughness at fracture. These exceptional improvements are attributed to the strong interfacial bonding between Gr and the epoxy matrix, as well as the tortuous path created by the well-dispersed graphene sheets, which effectively dissipates energy during crack propagation. The findings reveal a pronounce toughening and strengthening effects for epoxy composites from nitrogen doped graphene reinforcement paving the way for “green” and mechanically superior structural composites.

**Supplementary Information:**

The online version contains supplementary material available at 10.1038/s41598-025-11204-6.

## Introduction

Metallic corrosion is regarded as a worldwide and prevalent interfacial phenomena, which is one of the major issues confronting the steel industry^1^. Consequently, robust and enduring remedies are required to facilitate addressing the metallic corrosion obstacles^1,2^ Epoxy resins are considered the main chronic types of organic protective coatings for corrosion control and alleviation in the broad engineering sectors, including civil and mechanical engineering. This is due to their chemical resistance, high strength, and ease of processing^3^. Despite the diverse formations or treatments offered throughout epoxy resins, the common challenges are the voids or defeats generated all the way through the depth (e.g., caused by chemical shrink through the curing process of epoxy resins). These voids generate networked passes in subsequent levels of service, making them vulnerable to chemical attacks and resulting in premature failure or a shortened lifetime^4^. Hence, it is vital for the maturity of the recent anticorrosion manufacturing. To explore novel anticorrosive materials and/or coating systems which fulfill the requirements of protecting the metal from the corrosion or minimizing corrosive damage^2,5,6^.

Generally, Organic coatings’ anti-corrosive properties are enhanced by using of functional fillers. This leads to shield the organic coatings against corrosion right throughout three essential mechanisms. The first is barrier filler, which works by delaying the diffusion path of corrosive chemicals; the second is inhibitive filler, for instance phosphates and chromates that hinder the corrosion process which leads to rate reduction of the cathodic or/ anodic reactions; and the third one is sacrificial fillers, that includes zinc. Zinc dust is a corrosion-active filler which corrodes sacrificially against the metallic substrate, thus giving cathodic protection. Platelets with high aspect ratio, like clays and any other two-dimensional (2D) substance^7,8^are frequently used as barrier fillers. These barrier fillers may generate a structure resembling a brick wall, which delay corrosive species’ diffusion throughout the coating that force them to adopt a tortuous path, resulting in good gas and moisture barrier qualities^3^.

Graphene-based materials, like graphene nanoplates, graphene nanosheets and graphene oxide have demonstrated strong anti-corrosive characteristics when used in organic coatings^9^. This is due to its fascinating properties, their unique 2D structure, have high aspect ratio, theoretical high surface area of 2630 m^2^/g, complete impermeability, inert nature to oxidation, high optical transmittance, excellent electrical conductivity, high thermal conductivity, lightweight, outstanding chemical stability and mechanical capabilities, which marvelously endows the extra physical features such as high strength, good toughness, excellent optical properties, and thermal conductivities, making it the most ideal anticorrosion material. Literature review has demonstrated that commercial graphene, graphene oxides and other similar commercial derivatives could provide a great barrier against through-coating transport of chemical diffusion (e.g., Cl^-^, OH^-^ or O_2_)^6^potentially for enhancing corrosion mitigation and control of metallic structures^4,10,11^ These investigations demonstrated that coatings enhanced with graphene nanoparticles at concentrations ranging from 0.5 to 1.0 wt % may improve corrosion resistance.

Nitrogen-doped graphene oxide (NGO) serves a dual function as both a nanocontainer for corrosion inhibitors and a compatible filler that enhances the corrosion resistance of epoxy coatings. Nitrogen doping of GO is employed to enhance its electrochemical properties by incorporating nitrogen atoms into the carbon honeycomb lattice. This doping process is typically achieved through hydrothermal treatment a cost-effective and accessible method in the presence of nitrogen-rich precursors such as melamine, urea, or dopamine. During this process, certain C–C bonds are replaced with N=C, N–C, and C–N–C bonds, resulting in the formation of new active sites. Beyond corrosion protection, NGOs have also demonstrated broad applicability in fields such as sensors, solar cells, batteries, supercapacitors, pollutant adsorption, and electrocatalysis^12^. Keramatinia et al.^12^. reported a study of An epoxy composite reinforced with 0.2 wt% nitrogen-doped GO@Zn nano-layers was developed using hydrothermal treatment. The composite showed improved mechanical performance with a tensile strength of 46.48 MPa and 61.5% elongation at break, The study employed the traditional, time consuming methodology which relies on toxic oxidizing agents for synthesis GO, modified Hummer’s method, followed by hydrothermal process for preparing N-GO. Zhang et al.^13^ reported the use of silk nanofibers as a biomaterial to facilitate the preparation and dispersion of graphene; however, the method involved harsh experimental conditions. Therefore, the development of a natural, environmentally friendly biomass-based dispersant that can serve as a green and facile alternative to synthetic dispersants is highly desirable.

Regardless of the efforts and accomplishments achieved in this subject, the extant literature on graphene-reinforced coatings focuses mostly on electrochemical properties. Electrochemical strategies have been extensively employed to investigate the effect of graphene and graphene-related compounds on the anticorrosive characteristics of epoxy coatings applied to steel substrates. Nevertheless, electrochemical results cannot be used to forecast the real-world effectiveness of coatings in corrosive conditions since their physical significance is difficult to comprehend^3,4^. Thus, a more extensive assessment of the coatings, including tensile strength, ultimate strain (ductility), ultimate toughness, toughness at break, abrasion properties, and long-term performance, could be immensely useful in gaining a deep grasp of graphene-based coatings. Hence, in the current study, the corrosion resistance behavior and mechanical performance of epoxy coatings enhanced with novel, eco-friendly nitrogen-doped graphene nanosheets (Gr), derived from chitosan, were systematically investigated. The study employed salt spray corrosion testing, and static contact angle measurements. Coating adhesion was quantitatively assessed using the pull-off test to evaluate resistance to delamination and corrosion. Additionally, several durability and mechanical performance parameters were examined. Thus, the specific aims of this study are to: (i) develop an environmentally friendly and cost-effective synthesis route for nitrogen-doped graphene (Gr) using chitosan as a sustainable precursor, (ii) incorporate ultra-low concentrations (< 0.04 wt%) of NG into epoxy coatings and evaluate their dispersion behavior and structural compatibility, (iii) investigate the durability characteristics of the coatings, including impact resistance, bending flexibility, and surface hardness, (iv) quantitatively assess the coating adhesion using the pull-off test to evaluate resistance to delamination and corrosion, (v) characterize the interfacial bonding between NG and the epoxy matrix, as well as the adhesion strength at the steel/coating interface. (vi) investigate the corrosion resistance performance of NG/epoxy coatings through accelerated salt spray tests and static contact angle measurements, (vii) assess the mechanical enhancement imparted by NG, including tensile strength, elongation at break, toughness, and impact resistance, (viii) elucidate the mechanisms underlying the synergistic improvement in both corrosion protection and mechanical properties due to Gr reinforcement, (viiii) demonstrate the multifunctionality of the developed coating as an eco-friendly, long-lasting, anti-friction, and corrosion-resistant surface layer suitable for sustainable applications. Our findings demonstrate that the as-fabricated product is an eco-friendly, surface coating that is long-lasting, anti-friction, and behaves as a corrosion barrier for a sustainable environment.

## Experimental

### Materials and synthesis of graphene (Gr)

#### Materials

CS ((C6H11NO4)n) of a small molecular weight of 100,000–300,000 g/mol, cetyltrimethylammonium bromide (CTAB, 99.5%) and the ammonium hydroxide (NH_4_OH, 33% NH_3_) acquired from ACROS Organics™, WINLAB, UK, and Sigma-Aldrich, Europe, respectively. All chemicals were of analytical grade and were used without extra purification. Throughout the synthesis, the aqueous medium was double-distilled water.

#### Solvothermal synthesis of N-doped graphene (Gr)

Gr was synthesized according our previous study^14^. Briefly, chitosan was firstly dissolved in 100 ml of 40% (v/v) acetic acid. After that, CTAB powder was dissolved in it. With a volumetric percentage of 1/10, a small amount of milliliters of ammonium hydroxide solution were added to the entire mixture. The entire recipe was then enclosed in a stainless steel autoclave system lined with Teflon. with a vapor/liquid volumetric ratio of 3/2 and thermally treated in an oven for 4 h at 270° C. Here, CTAB/Chitosan weight ratio of 0.3 have been adjusted. The autoclave system was then subsequently cooled to room temperature prior to be opened to collect the finished product. A shinny fluff black solid formed in the Teflon tube. Finally, the graphitic product was filtered, washed multiple times with distilled water, and dried for two days in a vacuum oven at 70 °C. the sample code for graphitic sample is Gr.

#### Microstructural characterization of synthesized Gr

X-ray diffraction (XRD) was employed to identify and depict the produced powdery sample’ crystalline structure. XRD pattern was generated by Shimadzu XRD-7000 diffractometer (30 kV, 30 mA, Cu Kα + Ni-filtered radiation, λ = 0.15406 nm; Shimadzu Corporation, Tokyo, Japan). The analysis was conducted over a 2-hours, through a scanning rate of 4°/min and with scanning step of 0.02°. Fourier Transform Infrared (FTIR) spectroscopy was utilized to qualitatively evaluate the oxidation state of graphitic material by means of a Shimadzu FTIR-8400 S spectrometer (Shimadzu Corporation, Kyoto, Japan). The spectrum was collected all over a wavenumber range of 500–4000 cm^− 1^, through a resolution of 4 cm^− 1^ and a total of 25 scans. Raman spectroscopy is extensively recognized as an effective nondestructive device for investigating carbon nanostructures. It makes a distinction of single layer graphene or/ nanotubes from multilayer graphene or/ nanotubes depended on in-plane (lateral) crystallite size, crystal order/disorder, and the rank of chemical variation. By a 532 nm laser light, Raman spectrum of powdery sample was obtained on a Bruker SENTERRA Raman Microscope (Bruker Corporation). The wavelength variety was 500–4000 cm^− 1^. X-ray Photoelectron Spectroscopy (XPS) was performed to determine the atomic concentrations of carbon, oxygen, and nitrogen. XPS is a powerful tool for representing the doped N configurations of the graphitic lattice. The studies were performed using a Vario-Micro CHN elemental analyser (Elementar studies System GmbH, Langenselbold, Germany) and Specs Surface Nano Analysis GmbH Specs lab prodigy version 4.89.2-r104748 2022, Berlin, Germany.

### Materials and preparation of epoxy-based composite coating

- Dispersion Method and Mixing.

We utilized a two-step dispersion process consisting of ultrasonication followed by mixing. Initially, graphene nanosheets of various content were properly stirred and mixed for 2 h into xylene mixture of isomers as a solvent (was acquired by PIOCHEM), by an ultrasonic device with ultrasonic power of 150 W (ISOLAP, laborgeräte GmbH) to further exfoliate and evenly disperse the graphene nanosheets. Subsequently, the mixture is gradually poured into 60 g epoxy resin ARALDITE (PY 1092 style) and lastly 30 g curing agent (HY 1092) was immediately mixed for 30 min into the mixture. The epoxy resin and curing agent were gained by (Huntsman Corporation).

- Coating application and curing

**Fig. 1:**
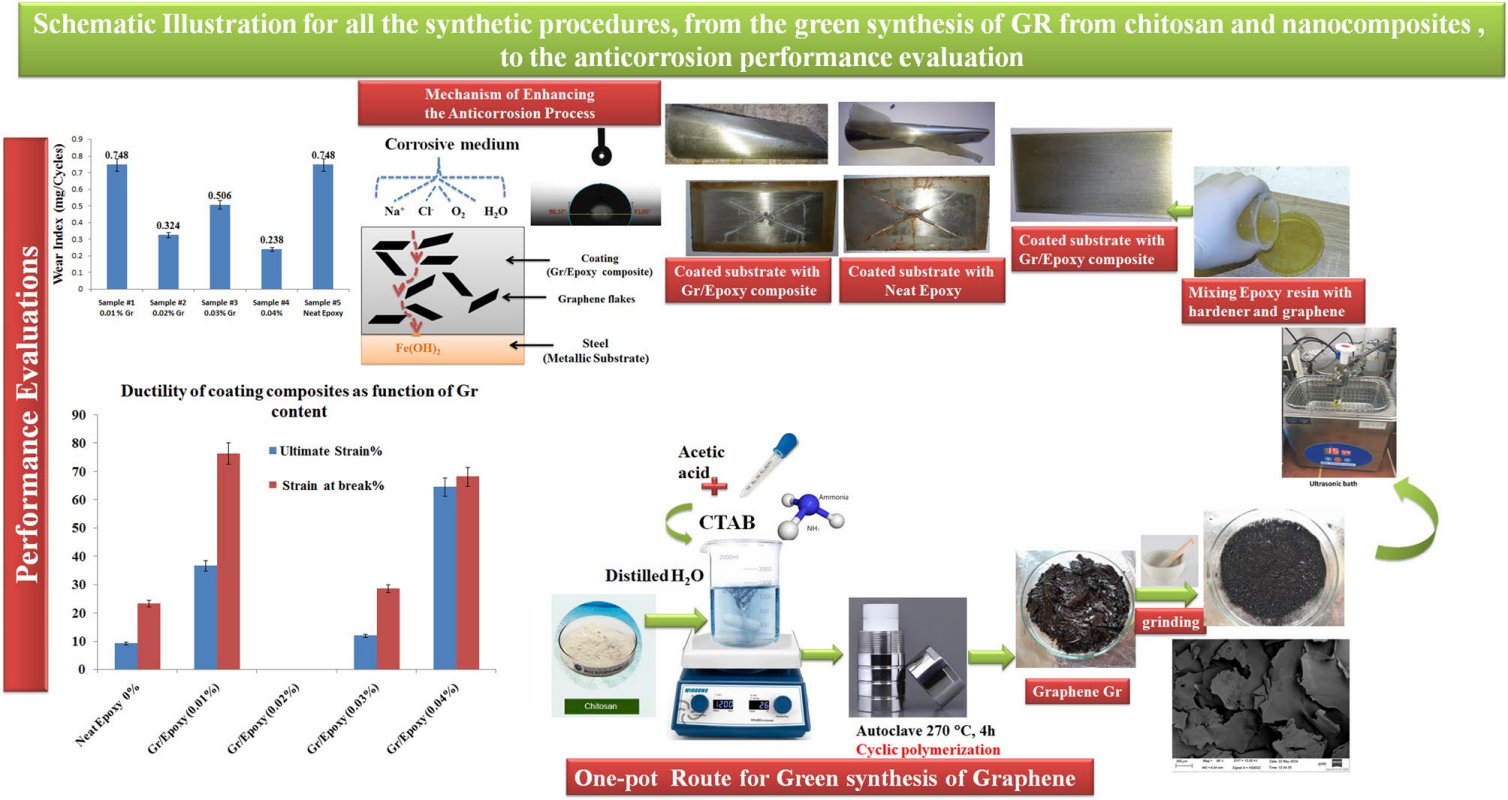
Schematic illustration for all synthetic procedures.

The carbon steel panels (size: 200 × 100 × 1 mm) were utilized as the substrates. The surface of the panels was re-polished with a metallographic abrasive paper. Prior to spraying, the substrate surface was washed with surfactant soap water, then rinsed and cleansed with de-ionized water, and lastly followed by washing with iso-propanol (purchased from CARLO ERBA). After applying the coating on the substrates, the prepared coatings were cured for seven days at room temperature. In according with the mentioned above method, five samples with varied graphene nanosheet content are shown in Table 1. The all preparation steps are depicted as a schematic illustration in Fig. 1.

**Table 1:** The chemical composition of the five samples.

Sample No.	#1 0.01%	#2 0.02%	#3 0.03%	#4 0.04%	#5 0%
Graphene (g)	0.01	0.02	0.03	0.04	0
Epoxy Resin (g)	60	60	60	60	60
Solvent (g)	17	17	17	17	17
Curing agent (g)	34	34	34	34	34

### Characterization and performance evaluation of composite coatings

The dry thickness of the Gr/epoxy composite coatings was exactly measured by an electronic coating thickness gauge (Elcometer 345 thickness meter (UK)). Ten readings were made crosswise the coating’s surface. Coating Hardness by pencil test, Shore A and Shore D harness are carried out according the standard test methods D3363 and ASTM D2240, respectively. The test assesses the penetration of a specified indenter into the material under specified force and time constraints.

#### Corrosion resistance tests

Accelerated durability test, (Salt Spray Test)

The long-term endurance of the as-fabricated composite coatings was assessed by an accelerated durability test, according to Salt Spray Test -ASTM standard B117 by a spray cabinet (C&W Specialist Instruments, SF/MP/AB100, UK). One of the most frequent employed accelerated corrosion test is the neutral salt spray experiment. The salt fog in the marine atmosphere is composed of droplets and chloride. The chloride ion is extremely corrosive and penetrable. The samples are located in the salt spray testing box, and the experiment solution is atomized and regularly distributed all the way through the coating’s surface. The test surroundings included a 5 wt% NaCl aqueous solution. The sample’s opposite facade was attached by a waterproof adhesive glue. Subsequent, the front surface was cross-knife and the specimens were placed in their respective beakers. The sample surfaces were maintained at 35 °C, in areas with high humidity, examined at various times, 385, 720 and 4320 h.

#### Evaluation of the physio-mechanical properties

- Bending and Impact resistance tests

Two distinct types of experiments were performed to demonstrate the effects of graphene loading on the coatings composites flexibility, bending test and impact resistance. Regarding the bending test, The coated substrate was twisted on a conical shaped mandrel (a Sheen Ref. 809 mandrel bending device (UK)) to analyze the elasticity of the coatings in accordance with ASTM D522 ^15^. The mandrel’s diameter ranged from 3.1 to 38 mm. Impact resistance was executed to evaluate the resistance of the fabricated coatings samples to sudden deformation by a falling weight. The test was carried out by a An apparatus of Sheen Ref BG5546 (UK), for examining the coatings’ impact resistance according to ASTM D2794-04 standard^16^. The whole weight and indenter was elevated 1 inch in the testing tube and permitted to fall on the coated substrate, and the falling weight test was repeated with height increments until a crack in the coating was detected. The height at which the coating cracks was measured, and the test was repeated five times each slightly above, slightly below, and at the recorded height, in accordance with the ASTM standard. The elevation at which the coating cracks in all five tests is described as the coating’s impact resistance limit to fast deformation.

- Adhesion Test.

The coating’s adherence to the substrate is determined via the test protocol for the pull-off strength of coating utilizing a potable adhesion detector in compliance with ASTM-D4541^17^, as a quantitative technique of measuring the coating’s corrosion resistance.

- Abrasion resistance and wear behavior

The coatings’ abrasion resistance was assessed using the Taber abraser procedure according to ASTM D4060, in which the samples were rotated at 72 rpm for 500 and 1000 cycles under a weight of 1000 g. The mass loss was then used to compute the abrasion resistance according the form (Eq. 1):^4^

1 $$\:Mass\:loss={M}_{intact\:coating}-\:{M}_{damaged\:coating}$$

where *M*_*intact coating*_ represents the weight of the initial intact coating (mg) and *M*_*damaged coating*_ represents the remaining mass after the rotated cycles (mg). The wear index, WI, was employed to get input on the abrasion resistance of the as-prepared new coating by the form (Eq. 2):

2 $$\:WI=\frac{{M}_{intact\:coating}-\:{M}_{damaged\:coating}}{Number\:of\:cycles\:of\:abrasion}$$

where mg / cycle is the WI’s unit. The new coating’s abrasion resistance increases as the wear index decreases. Higher abrasion resistance could safeguard the coating and its related substrate from the detrimental impacts of abrasive conditions that are common in civil structures.

#### Surface wettability

- Contact angle and hydrophobicity

The hydrophobicity of the neat epoxy coating and Gr/Epoxy composites coatings were investigated via the analysis of water contact angle and studies the non-wettability of the surface of the coated specimens according to ASTM D7490 and using a contact angle goniometer (Attention Theta Optical Tensiometer, Biolin Scientific Company, Finland). Water drops of 0.1 mL were carefully placed on the substrate with a micro-liter syringe. The obtained water contact angle was the average value of ten measurements.

#### Mechanical performance of composite coatings

Mechanical characteristics of the coatings were determined using the tensile test utilizing Shimadzu’s EZ-X tester with a displacement control of 1.0 mm/min according to ASTM D638. The tests that generated Young’s modulus, tensile strength, ultimate strain (ductility), elongation at the break, ultimate toughness and toughness at break, were assessed for deep understanding the damage resistance of the as-fabricated new coatings.

#### SEM of composite coatings

The fracture surface of the samples after tensile testing was cut out and gold coated before being submitted to SEM microstructural analysis.

#### Chemical structure study of composite coatings

Fourier-transform infrared (FTIR) spectroscopy was employed to analyze the composite coatings, providing insights into the chemical structure and confirming the formation of characteristic chemical bonds between the epoxy matrix and the incorporated nanofillers.This analysis supports the interpretation of the observed mechanical performance and other related physical properties.

## Results and discussion

### Confirmation of graphene structure and composition

#### XRD

**Fig. 2:**
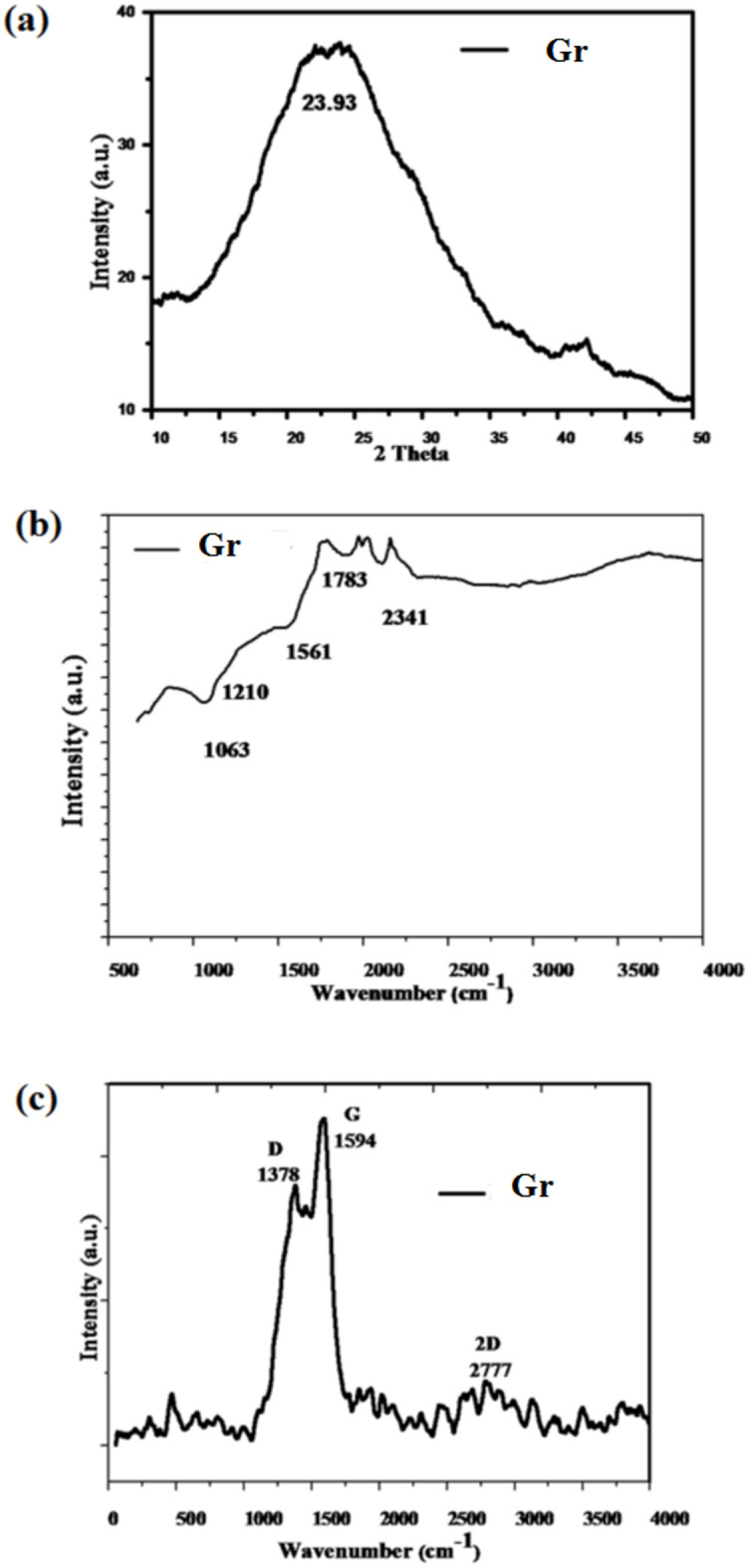
**a** XRD patterns, **b** FTIR spectrum and **c** Raman spectrum of solvothermally synthesized graphitic material Gr.

Figure 2a displays the XRD patterns for powder sample Gr. A wide peak is noticeable between 2ϴ=20–30°. This beak, seen at 23.93◦ with interlayer distance 0.37156 nm, is associated with (002), the structure’s plane of graphitic materials^18^JCPDS No. # (75-2078). These values are very close to recorded value of graphene; 26◦ and 0.34 nm^19,20^. The band’s broadness indicates the exfoliation of graphitic structure. Tang et al. described a feasible strategy for elucidating the bottom-up layer-by-layer construction of graphitic structure in their research on glucose polycondensation to produce graphene oxide^21^. Here, in brief under solvothermal environment, chitosan molecule, as a sort of amino polysaccharide molecules, constantly dehydrate and undergo cyclic polymerization, generating a monolayer of graphitic structure doped with nitrogen The second graphitic layer is consequently formed, with the previous layer acting as a substrate, resulting in a bi-layer graphitic construction. Afterwards, the tri-layer and multilayer graphitic structures can be produced. It is worth mentioning that at temperatures beyond 200 °C, ammonia as a structure directing reagent and/or CTAB begin to partially decompose, while polysaccharides cyclically polymerize to create the N-RGO network^20^.

#### FTIR

Figure 2b shows the FTIR spectrum of Gr. The presence of an IR absorption peak at 1561 cm^− 1^, which corresponds to the in-plane C = C vibration, demonstrates the sample’s graphitic characteristics in its FTIR spectrum. It is an inherent characteristic of the sp^2^ graphitic materials^22^. The sample’ FTIR spectrum shows band related to oxygen-containing functional group, showing remaining oxygen in the graphitic structure. Gr exhibits band for C–O stretching vibration at 1061 cm^− 123^. Moreover, sample’ FTIR spectrum illustrates bands associated with N atoms/ amino groups; tertiary amine, CN stretching vibration at 1210 cm^− 1^, nitrile C≡N and/or isocyanate −N=C=O at 2340 cm^− 1^ and NH bending of primary amine at 1783 cm^− 123^. Further, the functionalization of the graphitic framework of the sample has been validated by the emergence of the N-CH3 stretching band at 1220 cm^− 1^, which is found in the sample’ FTIR spectrum^24,25^.

#### Raman analysis

Figure 2c displays the Raman spectrum of the Gr. The spectrum reveals graphene’s Raman G and D bands, which are attributed to in-plane vibrational mode of aromatic sp^2^ carbon (C = C) and the lattice disorder of the graphitic structure, respectively^26^ .The 2D band is also has been observed, it is a second-order Raman characteristic, referring to crystalline graphitic material and responsive to the aromatic C-structure. The data are tabulated in Table 2. Moreover, The intensity ratio of I_D_/I_G_ can also be used to quantify a specific sort of sp^2^ lattice structure order/disorder known as sp^2^-edge order/disorder, which is disorder that is localized around the edges of sp^2^ crystalline particles (domains) but not within them. Thus, it is indicative of the propagation of sp^2^-edge disorder within graphitic powder i.e., disorder localized at the edges of sp^2^ crystallites (domains) but not the interior of them^19,20^. Additionally, to further study the structure ordering of the graphitic substance, the lateral size of the aromatic sp^2^ domains (La) which is expressed by average in-plane crystallite size has been estimated by the Tuinstra and Koenig relation^19,20^, which is declared below (Eq. 3):

3 $$ La = C(\lambda)/(I_{D}/I_{G}))$$

where the coefficient C(λ) is a wave length dependent pre-factor (~ 4.4 nm)^19,20^. The improvement of the structure ordering in the graphitic material is advance established from the low ratio of I_D_/I_G_ and enlarge of the lateral dimensions.

**Table 2:** Raman data of the solvothermal graphitic sample Gr.

Sample code	Position of bands (wavenumber (Cm^− 1^))			Bands intensity		Intensity ratio	L_a_ (nm)
	D	G	2D	I_D_	I_G_	I_D_/I_G_	
Gr	1378	1594	2777	15.62061	18.26437	0.85	5.17

#### XPS analysis

The chemical structure of Gr was further investigated in detail using X-ray photoelectron spectroscopy (XPS), a powerful analytical technique capable of determining both the elemental composition and the chemical bonding states within a sample. XPS was employed to confirm the successful incorporation of nitrogen into the graphene framework and to identify its bonding configurations. The XPS survey spectrum of Gr Figure ***S1***, included as Supplementary material, revealed the presence of carbon, oxygen, and nitrogen, with atomic concentrations of 77.65%, 13.66%, and 8.69%, respectively. These findings confirming that nitrogen atoms were successfully integrated into the sp² carbon network of the graphene sheets. Notably, the nitrogen content of 8.69 at% is considered relatively high compared to values typically reported in the literature^27,28^underscoring the efficiency of the employed synthesis method. No other elements were detected, indicating the high purity of the synthesized graphitic material. High-resolution deconvolution of the C 1s and N 1s spectra Figure S1a and b, respectively) was carried out using standard XPS peak fitting software, and the resulting peak assignments were consistent with previously reported literature. The C 1s spectrum exhibited characteristic peaks corresponding to various functional groups: sp²-hybridized carbon (C=C) at 285.02 eV (62.57%), C=N and C=O groups at 287.09 eV (5.77%), and carboxyl (O–C=O) groups at 288.63 eV (7.40%)^27,28^.

The deconvolution of the N 1s spectrum revealed two distinct nitrogen bonding states, typical of N-doped carbon materials. The dominant species was pyrrolic nitrogen at 399.4 eV, accounting for 97.72% of the total nitrogen content, while a minor contribution of graphitic nitrogen was observed at 405.05 eV (2.28%). These results further support the successful doping of nitrogen atoms into the graphene lattice rather than their presence as merely surface-bound functional groups. Collectively, the C 1s and N 1s deconvolution profiles, together with FTIR analysis, confirm that Gr is a low-oxygen, nitrogen-doped graphene material with high purity and structural integrity. The incorporation of nitrogen into the sp² framework enhances the chemical functionality and potential reactivity of the graphene nanosheets for composite applications.

### Morphological analysis

#### SEM of Gr

**Fig. 3:**
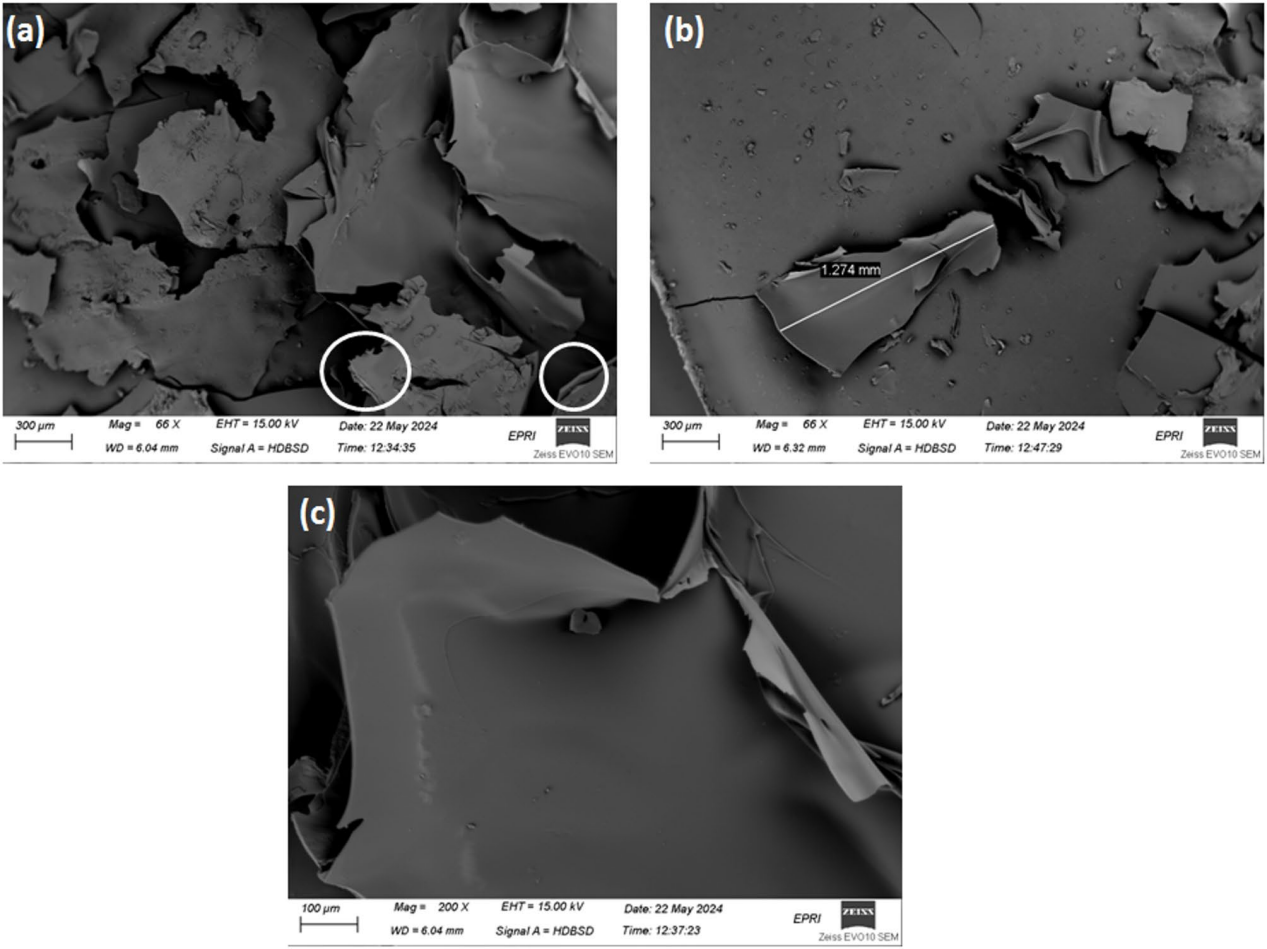
SEM micrographs of Gr (a, b) and its magnification micrograph (c).

The morphological features of the prepared sample Gr are depicted in SEM micrographs in Fig. 3a–c. The graphitic structure dominant in the Gr, which is presented in Fig. 3a, reveals exfoliated multi-layered thinner sheet architecture. These sheets have a flat structure with extremely extended wide average lateral dimensions more than 1.27 mm, as shown in Fig. 3b. Theses sheets are superfine appear similar to well separated smooth morphology with folding and wrinkled characteristics, as depicted in Fig. 3c. These observations evidence a distinctive superior surface area of the graphitic material, Gr. By increasing the CTAB dose as compared by the previously published work^14^there are significant increase in the lateral dimension from 500 μm to 1.27 mm. This could be explained in light of thermal decomposition of the CTAB molecules under the effect of nature of solvothermal process under the autoclave pressure and temperature.

In the previous work, it was found that increasing the temperature from 250 to 270 °C leads the spherical particles to diffuse into each other throughout open edges, as a final point producing a sheet-like structure^14^. As mentioned there, increasing the CTAB dose slightly increases the extent of hydrogen gas generation throughout the hydrothermal reaction system that results from thermal degradation of CTAB, whereas raising the hydrothermal synthesis process temperature greatly boosts it. This, in turn, amplifies the collisions between the hydrogen gas ions and causes their internal energy to dissipate as heat. It is worth noting that Hydrogen has been recognized as an energy carrier element that is defined by high endothermic enthalpy of production of a hydrogen gas ion (1536.3 kJ/mol)^24^. As a result, any collision between two hydrogen- gas ions produces an excessive amount of dissipated heat, resulting in sintering of the graphitic spherical particles. When big groups of spherical particles fuse together, a sheet-like structure emerges, as shown in the SEM images. The enhanced heat dissipation through the hydrothermal reaction media causes several groups of spherical particles to sinter into a variety of sheet-like shapes. This is thought to be why the sheet design dominates over the spherical one in graphitic samples with higher CTAB doses and hydrothermal synthesis temperatures^24^.

### Coating performance evaluation

#### Corrosion performance

Accelerated durability test, Salt Spray Corrosion.

**Fig. 4:**
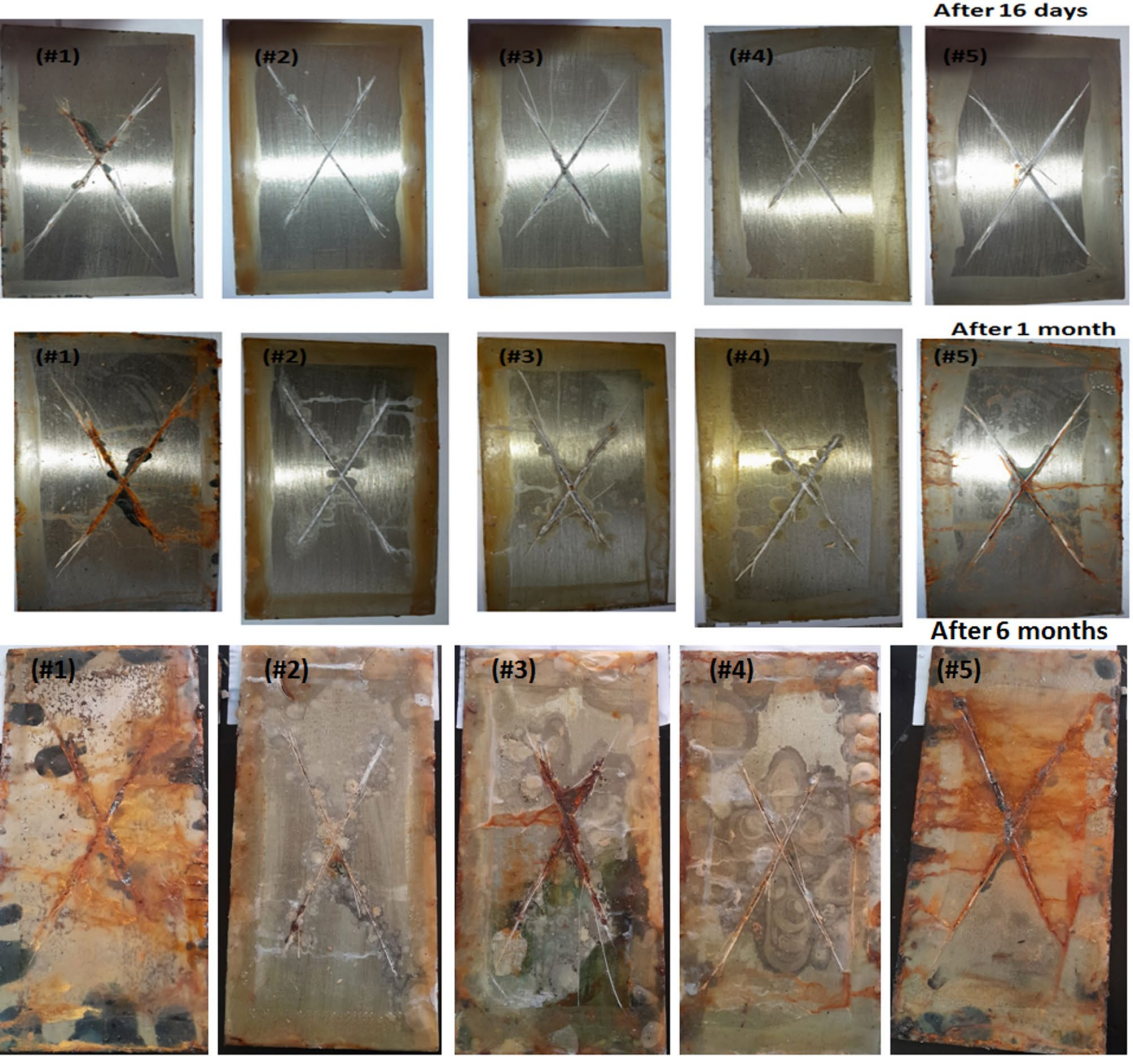
Macrophotographs of samples #1–5 in 3.5 wt-% NaCl aqueous solution at different exposure time.

Figure 4 depicts the macrophotographs of five samples that were subjected to the salt-fog environment for varying periods. The upper group in Fig. 4 consists of five samples that got subjected to salt-fog for 16 days; sample #5; the neat polymer and sample#1; the sample which containing the lowest content of graphene, their surfaces were somewhat started to be influenced. However, the other three samples containing graphene were not visibly degraded. The second group in Fig. 4 consisted of five samples that got exposed to salt-fog for 1month; samples #1 and #5’s surfaces were somewhat corroded. Nevertheless, the other three samples including graphene were not visibly degraded. The corrosion from the salt mist on the material’s surface was generated via the chemical reaction of an oxidative layer consisting of chloride ions that pass through the barrier of protection until it reached the interior metallic substrate. By gradually increasing the exposure time, The surface of the protective coating has changed into an activated surface, leading the metal to be slightly corroded. This is because the chloride ion contains a certain amount of hydration energy, that is merely adsorbed on the metallic surface and squeezed, substituting the oxygen within the chlorination layer. This intern converts the insoluble oxides into the soluble chlorides, transforming the passivation surface into an activated surface and causing the metal to be corroded^9,29^.

Upon extending the exposure duration to six months, the neat epoxy resin coating devoid of graphene and sample #1, which contains the minimal graphene content, experienced significant corrosion due to chloride ions. These ions rapidly go through The tiny pores or small cracks in the polymer covering on the metal surface, instigating a chemical reaction that led to the formation of bronzing-colored products. Those are blisters and rusting spots which mean corrosion and rust formation. As a result, the surface of samples subjected to salt-fog conditions exhibits extensive pitting corrosion, whereas the other three coatings were less effected in comparison to the other two samples#1 and #5, Fig. 4. This could be due to after subjecting to salt-fog conditions in the Cl^−^ ions solution for a long-term exposure, the epoxy coating failed for samples #5 and #1 due to penetration by Cl^−^ ions, O_2_, and H_2_O molecules. However, graphene-containing samples exhibit superior shielding properties, making them impenetrable to Cl^−^ ions, O^−^ molecules, and H_2_O molecules. This protects metal against corrosion^9,29^. Samples #2 and #4 have comparable result, show the best corrosion protect phenomenon. Hence, they are considered the best samples this could attributed to the good distribution and well dispersion of graphene within the polymer matrix. This leads to increase their surface area and the epoxy/graphene interfacial bonding, the steel/coating adhesion strength and water repellency. This intern, prevent the water and corrosive ions permeability and suspect to trapping them. This redound in reducing the rusting and blistering formation and enhancing the hydrophobicity and the coating-steel cohesion forces.

**Fig. 5:**
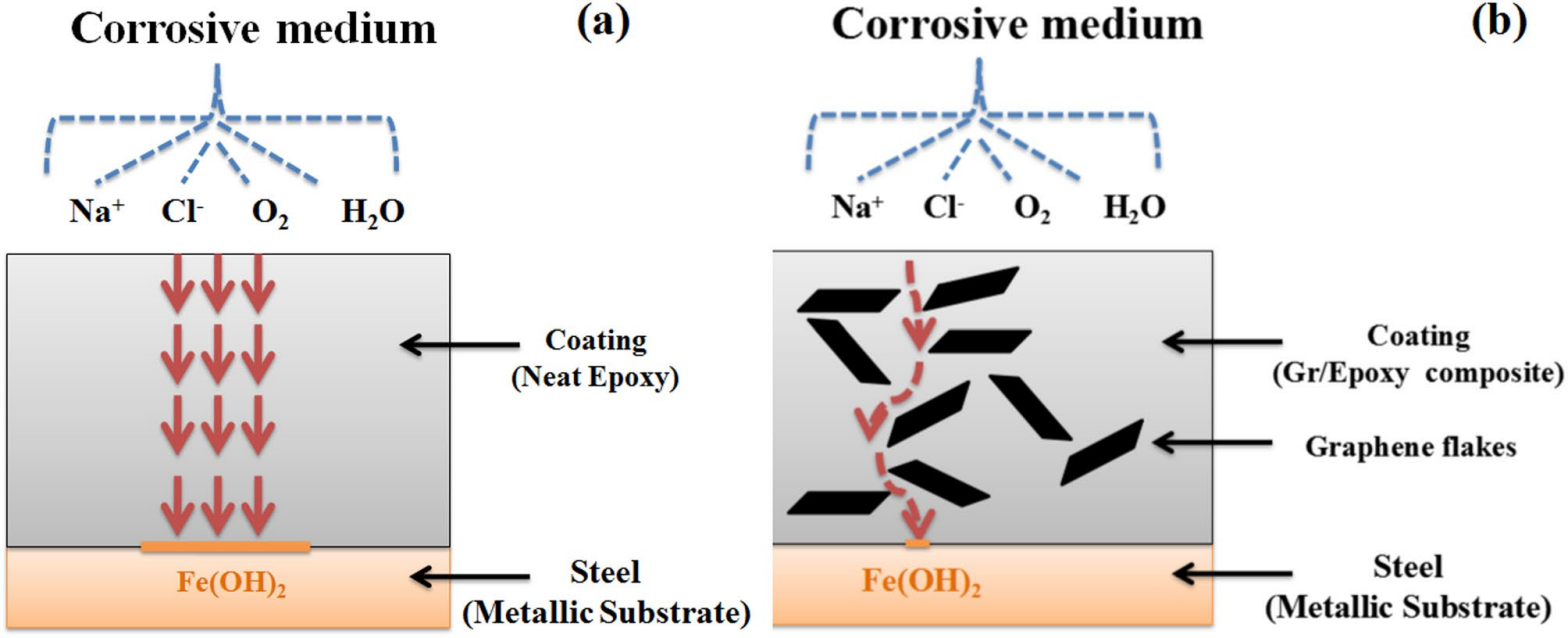
Schematic Representation of the corrosive agents passes through (a)neat epoxy coating, (b) Gr/Epoxy composites.

The suggested mechanism of the enhancement of corrosion protection process depend on understanding how the failure of epoxy coating occur. The initial stage involved water uptake in the film, followed by Cl^−^ ion diffusion through the coating^9^. The graphene nanosheets were inserted and arrayed in the epoxy matrix, serving as a highly effective barrier to capillaries holes diffusion and increasing the tortuosity of the O_2_ molecules’ diffusion channel, permeating the composite coatings. (see Fig. 5, depicts the schematic representation of the corrosive agents passes through). Although graphene increases molecular diffusion resistance in the coating matrix, H_2_O, O_2_, and Cl^−^ ions can still enter over time. The graphene’s convoluted path eventually enables the Cl^−^ ions to arrive at the metal-covering interface. After a prolonged period, the conductive character of the graphene nanosheets force electrons conduction which produced by an anodic reaction with a primary steel, resulting in a less corrosive effect.

#### Physio-mechanical performance

Impact, bending, abrasion, hardness.

**Bending and Impact Resistance Tests**.

Mechanical properties like elasticity and impact resistance are significant features of polymer coatings. Bending and impact resistance experiments were performed on epoxy and Gr/Epoxy composite coatings to assess the influence of graphene on flexibility and impact resistance. Figure S2, included as Supplementary material, displays bending and impact resistant results for various coatings. These findings show that the addition of graphene improves the flexibility of the epoxy coating, even as graphene loading elevates. Moreover, adding graphene improves the impact resistance of epoxy. No cracks upto 18 J were observed after performing the impact test for Gr/Epoxy nanocoating, which indicates high flexibility and durability of the coating (Table 3). The degree of improvement is related to the graphene loading. The observed influence of graphene on impact resistance could be due to increased toughness of the epoxy composites loaded with, which improves the epoxy coating’s resistance to abrupt deformation.

**Table 3:** Testing the mechanical performance for the unfilled Epoxy and Gr/Epoxy composite coatings and their standard deviations gotten from 3 different measurements

Properties	Sample#1	Sample#2	Sample#3	Sample#4	Sample#5
DFT (av.) (µ)	700	640	600	700	655
STDEV	±1.145	±0.536	±1.640	±1.478	±1.276
Adhesion Strength (µpa)	9.2	5.4	5.7	7.6	4.7
STDEV	±0.164	±0.102	±0.101	±0.369	±0.169
Impact Resistance (Joule)	3.6	18	18	12.6	3.6
STDEV	±1.26	±0.08	±0.93	±0.071	±0.91
Bending test (mm of cylindrical spindle)	< 5	< 5	< 5	< 5	Failed
Hardness (pencil)Test (N)	5	20	20	15	5
Hardness Shore A	106	107	88	108	104
Hardness Shore D	93	95	105	86	100
Abrasion Test ( at 500 cycles) Mass loss(mg) Wear Index	374 0.75	162 0.32	253 0.5	119 0.23	374 0.75
STDEV	±1.34	±0.093	±1.02	±0.08	±1.4

**Fig. 6:**
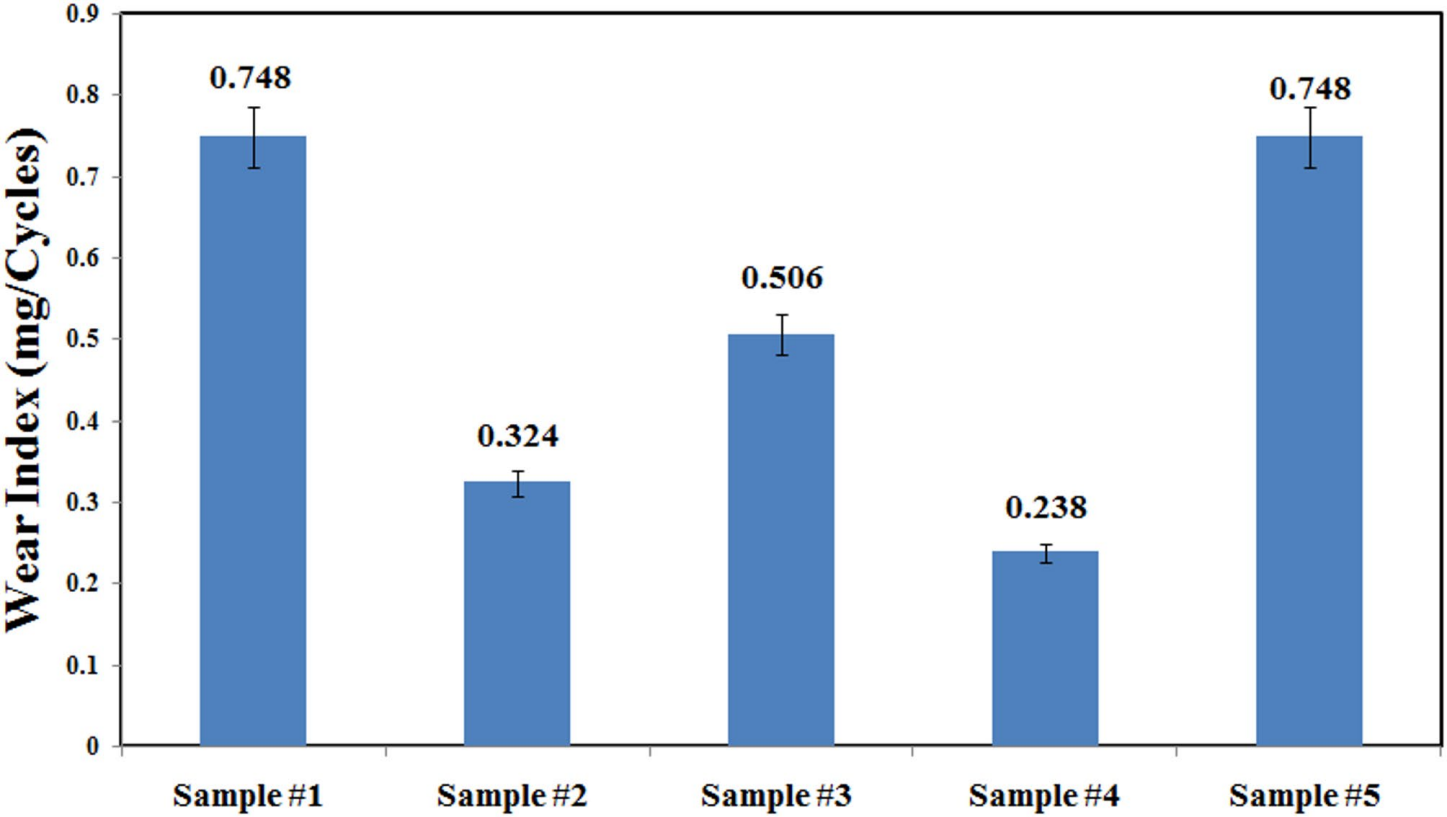
Wear indices of the tested samples after 500 cycles, with error bars and standard deviations gotten from 3 various measurements.

**Evaluation of Abrasion resistance** Another quantitative test of damage tolerance was the abrasion resistance of the substrate using the Taber abraser procedure according to ASTM D 4060. The test sample findings were reported as mass loss based on Eq. (3) or wear index based on Eq. (4), as shown in Fig. 6. It is obvious that the neat epoxy coated samples experienced the greatest mass loss, around 374 mg after 500 cycles. The sample#1 0.01 wt% Gr-Epoxy was matching to the neat epoxy, with a small abrasion resistance, as revealed in Table 3. Inclusion of graphene contents in samples #2, #3 and #4 ranging from 0.02, 0.03 to 0.04 wt % in the composite coatings revealed enhanced abrasion resistance by 58%, 33% and 70% at 500 cycles, respectively. Interestingly, samples with greater amounts of graphene, 0.04% Gr-Epoxy, maintained a modest mass loss when compared to pure epoxy. In order to determine for the rate of abrasion resistance as time goes on, the wear index was calculated based on Eq. (4) and expressed as Fig. 6, where y- axis represents the mass loss over cycles. A comparison of mass loss after 500 cycles indicated that composite coatings enhanced with graphene components were able to significantly minimize mass loss, with a minimal wear index of over 70% less, as compared to the plain epoxy, as shown in Fig. 6. For example, the neat epoxy, sample #5, exhibited a wear index of 0.748 after 500 cycles, whereas the 0.01% G-Epoxy, sample #1, stayed constant. Whereas, samples #2, #3, and #4 showed a significant reduction of 56%, 32%, and 68%, respectively, compared to the neat polymer. This 2D-additive material with its layered structure, enjoyed with low shear strength (weak layer-layer interaction), can avoid direct contact, lowering the friction coefficient and speeding up wear due to the rolling impact of nanofiller on the contact areas. Therefore, the use of graphene nanosheets produces higher effortless sliding and prevents metal-to-metal contact, which improves the abrasion resistance, and consequently lowering friction. The lower friction value was explained by electrons accumulated between the carbon atom and the attached Nitrogen atom, As a result, the repelling interaction between two contact sheets is minimized, as is energy generation. Furthermore, the adhesive capabilities of N-doped graphene were enhanced.

**Hardness Evaluation**.

The shore hardness assessments yielded identical results to the tensile tests, indicating a co-relationship between hardness and tensile strength as mechanical properties. The samples#4 and 2 showed the largest hardness values of 108 and 107 when compared with that of pure epoxy sample of 104.

#### Adhesion and durability performance

Epoxy and graphene anticorrosive coating (650 μm) is tested according to D4541 “pull-off adhesion test”. The experimental findings are depicted in the Figure S3, included as Supplementary material. The results of the pull-off test are shown as adhesives. The adhesion strength of the coating composites incorporated with four different concentrations of graphene was greatly improved compared with the neat epoxy adhesive. Sample #1 had the most adhesion strength, which resulted in the adhesion strength being increased from 4.7 to 9.3 MPa, Then the strength of sample #4 was 7.6 MPa.

#### Surface wettability

- Contact angle and hydrophobicity

The non-wettability of the coated samples was analyzed by detecting the contact angle which reflect the surface water hydrophibicity. The neat epoxy coating shows a contact angle of 62.01° ±1.6°, this is in a good agreement with the literature^30^. The contact angle increased as the graphene content increased in all the composite coatings as shown in Table S1 in supplementary materials. Based on the pre-evaluation of interfacial contact angles, a higher contact angle reflects reduced hydrophilicity and increased lipophilicity and hydrophobicity, which in turn promotes superior dispersion of the filler within the organic epoxy matrix and enhances interfacial compatibility. Among the evaluated formulations, the composite coating incorporating only 0.02 wt% graphene demonstrated the highest degree of hydrophobicity, exhibiting a 50% increase in contact angle (31.04° higher) compared to the neat epoxy. This enhancement suggests an optimized spatial configuration between the graphene nanosheets and the epoxy resin, effectively impeding the penetration of corrosive species. Moreover, π–π electron donor–acceptor interactions between the graphene surface and corrosive agents further contributed to the hydrophobic behavior. As a result, the coating exhibited significantly enhanced corrosion resistance. These findings underscore the pivotal role of graphene’s inherent impermeability in corrosion protection, serving as an efficient barrier against the ingress of aggressive media such as reactive gases, electrolytes, and acids. This highlights the potential of graphene/epoxy composite coatings as multifunctional materials for advanced structural applications, including corrosion-resistant protection in CO₂–Cl⁻ environments, particularly relevant to aerospace structures.

#### Mechanical performance

**Fig. 7:**
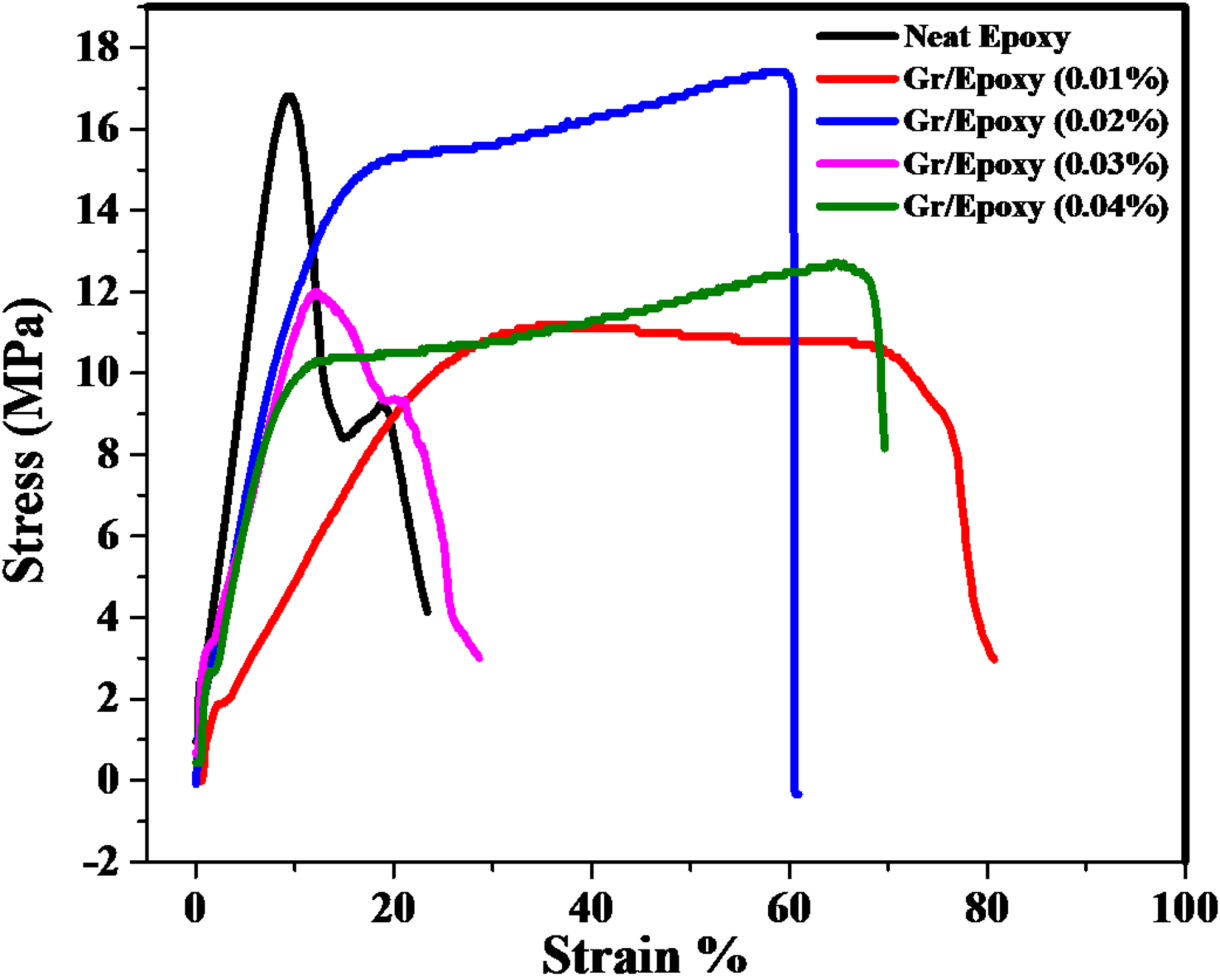
Tensile stress-strain curves of neat Epoxy and Gr/Epoxy coating composites.

**Fig. 8:**
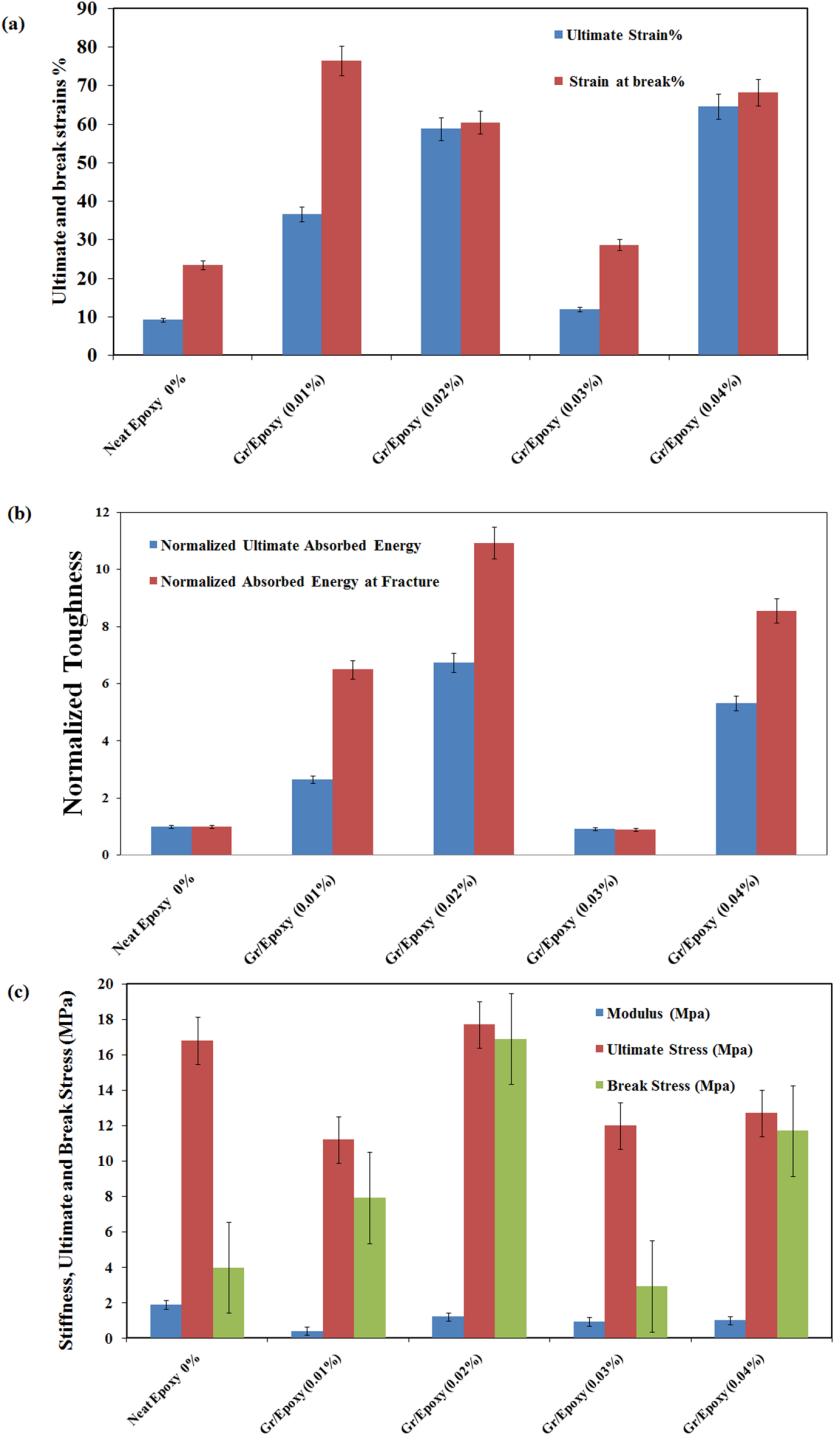
Tensile properties of the Gr/Epoxy coating composites as a function of Gr content. (a) Ductility, (b) Toughness and (c) Stiffness & Strength.

Understanding the mechanical characteristics associated with novel graphene-loaded composites can help evaluate their damage endurance in various environments. As a result, the tensile parameters of the composite coatings, comprising tensile strength, Young’s modulus, ultimate and fracture strains, and absorbed energies, that express the materials toughness, up to the ultimate and fracture points of neat Epoxy and Gr/ Epoxy coating composites, have been evaluated from the related stress strain curves, conferred in Fig. 7. The data are displayed versus Gr- content in Fig. 8. Generally, the mechanical properties were estimated here using the tensile test according to ASTM D2370-16 ^31^ as summarized in Fig. 8. Incorporation of Gr into epoxy has a significant improvement in the ductility even at ultra low content of Gr. It has shown that the graphene filler nanosheets suppressed cracks propagation within epoxy matrix with all the various filler content. The incorporation of Gr into the epoxy coating at ultra-low levels of 0.02 wt% and 0.04 wt% resulted in substantial enhancements in ductility; ultimate and elongation at break, with increases of 538%, 158%, and 603%, 191%, respectively.

The results have also shown that the loading Gr made tougher coating than the pure Epoxy coating. The addition of 0.02 and 0.04 wt% graphene filler resulted in a significant improvement in ultimate toughness and toughness at break by 573, 933% and 431,755%, respectively. The nanocomposites containing Gr would become more flexible than the neat epoxy coating, and the samples exhibited a significant increase in elongation. The dispersion of Gr sheets in the epoxy matrix consisted of a more homogeneous mixture, which would result in the increase in elongation at break and greater energy of fracture^32^. Moreover, (i) Gr sheets could provide rough and wrinkled surfaces to create larger interfaces; (ii) the wrinkled structure could prevent the detachment of nanofillers from the epoxy matrix. The characteristic structure had a great potential to keep the chains together. While, the elastic modulus and tensile strength values were slightly lower than that of the pure Epoxy for all samples except for the sample loaded with 0.02wt% of Gr. This could be a consequence of the large lateral dimensions of the graphene sheets. Larger sheets tend to bend; indeed it has been shown that Gr nanosheets adopt wavy or wrinkled structures, which may effectively reduce the modulus^33^. It has present a slight improvement in the tensile strength by 5.3% as compared with the neat epoxy. The superior mechanical performance of composite coating based on graphene over carbon nanotubes and any other nanofiller was due to their high specific surface area, better nanofiller matrix adhesion/interlocking arise from their 2-D flat geometry and their wrinkled (rough) surface^34^. Hence, the Gr/Epoxy composites with varying nanofiller loadings exhibit distinct mechanical responses—where *Neat Epoxy* demonstrates higher stiffness and stress-bearing capacity, making it suitable for structural applications requiring minimal deformation, while *Gr/Epoxy (0.01*, 0.02, 0.03 and 0.04%) displays greater strain tolerance and energy absorption, indicating its usefulness in impact-absorbing or flexible systems.

### Structure–performance correlation and mechanism.

- SEM of the composite coatings.

**Fig. 9:**
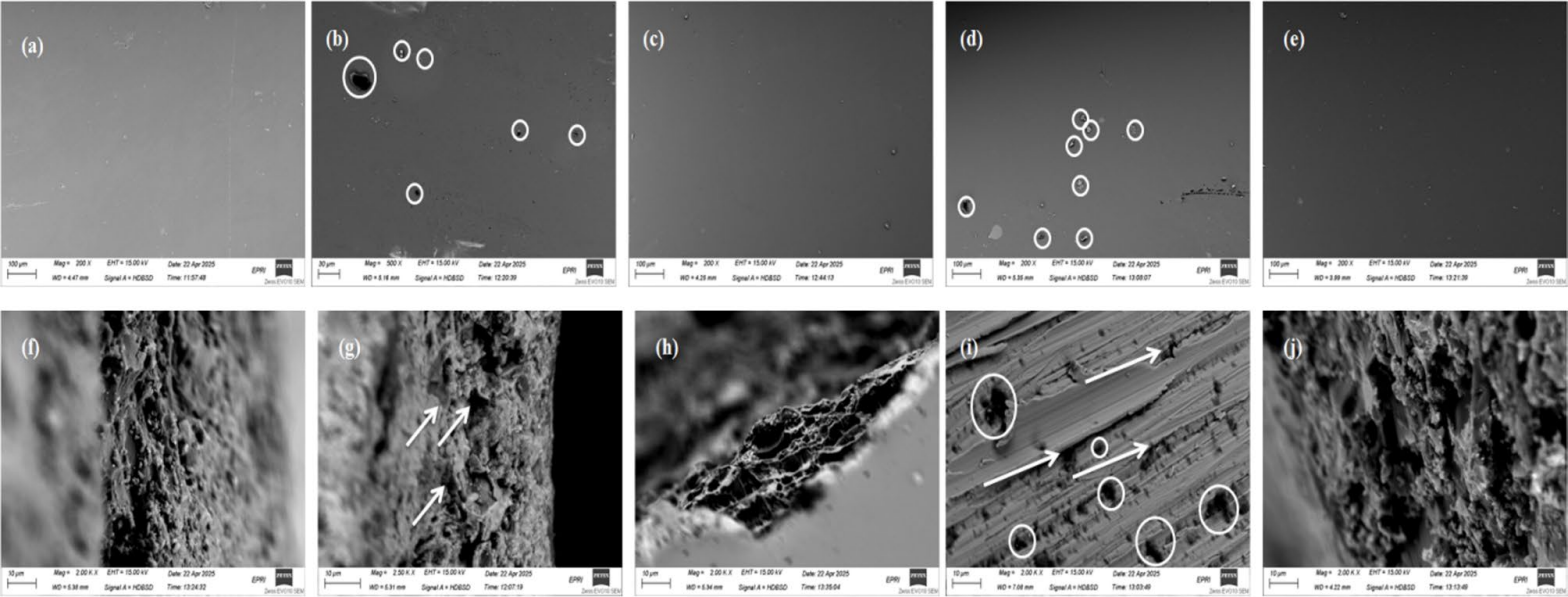
SEM micrographs of the **a** neat epoxy coating, **b** Gr/Epoxy (0.01%), **c** Gr/Epoxy (0.02%), **d** Gr/Epoxy (0.03%), Gr/Epoxy (0.04%), composites, and **f**, **g**, **h**, **i** and **j** the magnified images of their fracture surfaces, respectively

The surface and fracture micromorphologies of the neat epoxy and conposite coatings are shown in Fig. 9. More importantly, there are no defects or microcracks on these coating surfaces, which might result in better protection barriers to inhibit the permeable diffusion of corrosive media. The surface micromorphology, which depicted in Fig. 9a–e, show the SEM image of pure epoxy sample as shown in Fig. 9a, the surface was cleared and planed, even as certain graphene fillers flock together on the coated surfaces, meaning that some graphene sheets reassembled to form bigger particles during the combination with epoxy resin as shown in Fig. 9b,d of Gr/Epoxy (0.01%) and Gr/Epoxy (0.03%), respectively. whiles, a clear, darker and smoothed morphologies are depicted for Gr/Epoxy (0.02%) and Gr/Epoxy (0.04%) in Fig. 9c,e, respectively. the (a)neat epoxy coating, (b) Gr/Epoxy (0.01%), (c) Gr/Epoxy (0.02%), (d) Gr/Epoxy (0.03%), Gr/Epoxy (0.04%), composites, and Fig. 9f–j the magnified images of their fracture surfaces, respectively. The fracture micromorphology Fig. 9f–j reveal some accumlated graphene stakes on the fracture surface and a considerable number of micropores and microcracks caused by the pullout of graphene fillers, as shown in Fig 9g,i for Gr/Epoxy (0.01%) and Gr/Epoxy (0.03%), respectively. However, the fracture surface of Gr/Epoxy (0.02%) and Gr/Epoxy (0.04%) in Fig. 9h,j, depict no pores nor crack but embedded graphene sheets integrated with the epoxy. This implies that graphene sheets were evenly distributed and consolidated throughout the inside of composite coatings, and graphene has a good adherence with epoxy. Similarly, permeability dispersion of corrosive media can be efficiently inhibited. In addition, Gr/Epoxy (0.03%), has the largest pullout micropores. This phenomena could be explained by the fact that graphene sheets not dispersed well in Gr/Epoxy (0.03%),, resulting in bigger reassembling particles. This in a good agreement with the proposed explaination of why this sample showed a decline behavior in all mechanical parameters and all the physocimical properties. Furthermore, Gr/Epoxy (0.02%) and Gr/Epoxy (0.04%) considered the optimum of the study.

The selection of 0.02 wt% as the optimal graphene loading is based not only on the experimental results but also supported by theoretical and mechanistic considerations specific to the nature of two-dimensional (2D) nanomaterials and their interaction with polymer matrices. Especially, here in our study we prepared Nitrogen-doped graphene (Gr) exhibits a very high aspect ratio, with lateral dimensions extending beyond 1 mm while maintaining atomic-scale thickness (~ 0.3–1 nm). This geometrical anisotropy leads to a strong tendency for restacking or agglomeration, especially in viscous matrices like epoxy resins. As the aspect ratio increases, the effective percolation threshold for functional performance (mechanical or electrical) is reached at significantly lower weight fractions compared to spherical or 1D fillers. However, beyond a critical concentration, the risk of poor dispersion increases dramatically due to the viscous drag and limited shear forces during mixing, which hinders homogeneous distribution and promotes filler aggregation. At loadings higher than 0.02 wt%, we observed increasing evidence of graphene sheet agglomeration under SEM and reduced interfacial contact efficiency. Such agglomerates act as stress concentration points, which negatively impact the mechanical integrity and barrier performance of the coating. Furthermore, poor dispersion compromises the tortuous path effect necessary for corrosion resistance enhancement. Thus, 0.02 wt% was found to be the optimum balance high enough to establish effective filler–matrix interaction and network formation, yet low enough to avoid agglomeration issues that compromise mechanical and corrosion performance. This rationale aligns with theoretical predictions for the percolation behavior and interphase saturation of high-aspect-ratio 2D materials in polymer systems.

- Chemical Structure of composite coatings

**Fig. 10:**
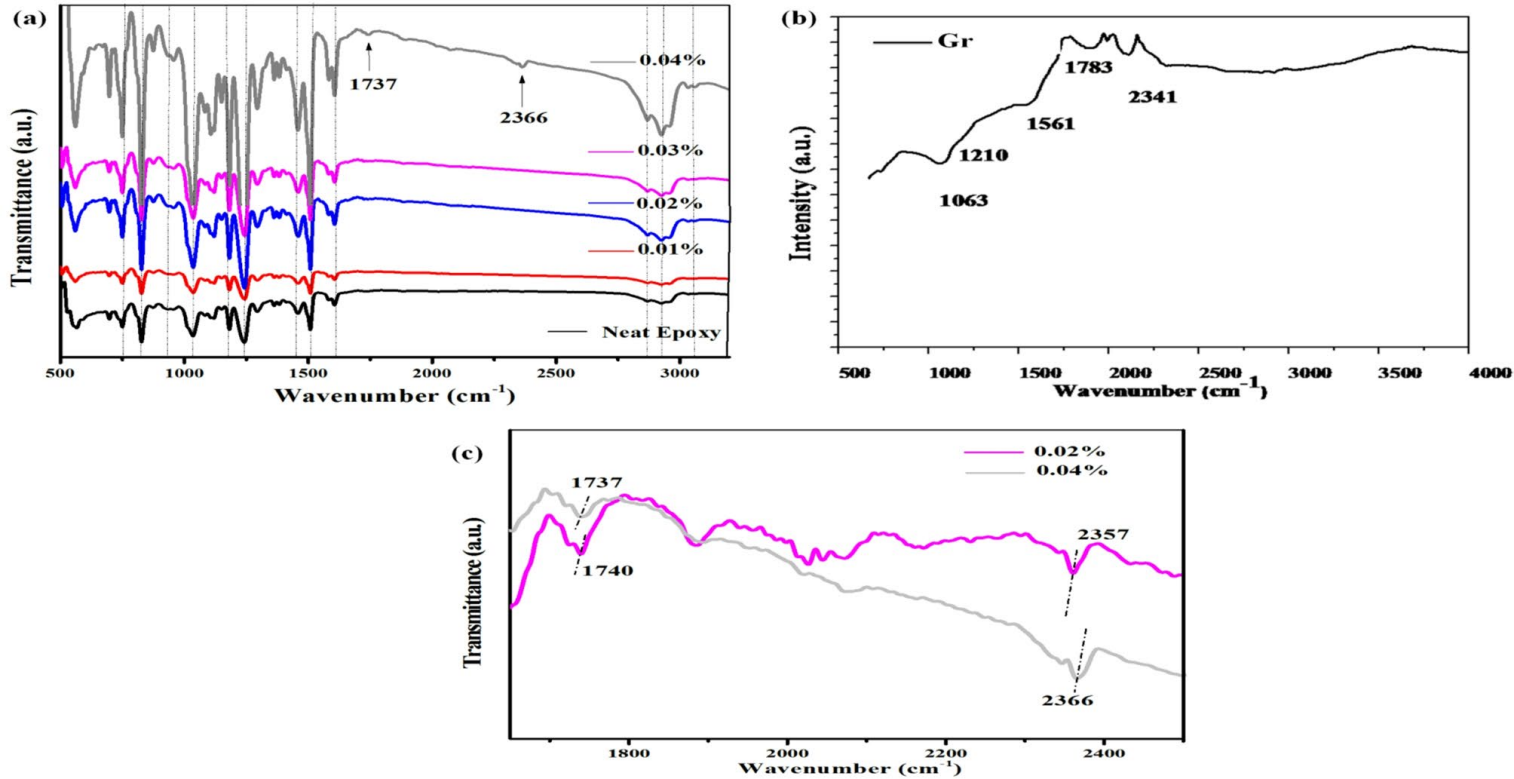
FTIR spectra of the Gr/Epoxy coating composites as a function of Gr content. **a** FTIR spectrum of Gr (**b**) and Zoom in of the FTIR regions **c** 1650–2400 cm^−1^ Gr/Epoxy coating composites with Gr content 0.02 wt% and 0.04 wt%.

In order to further understand the performance of the as-fabricated coating composites, the chemical structure of the coating composites has been studied via FTIR spectroscopy. The FTIR spectra of the neat epoxy and of the Gr/Epoxy coating composites as a function of Gr content are illustrated in Fig. 10a. Regard to the EP coating spectrum, the distinctive absorption peaks around 2920 and 2865 cm^– 1^ are caused by the C–H asym./sym. stretching vibrations. The absorption peaks corresponding to = C–H and C = C in the benzene ring appear at 3065 and 1600 cm^– 1^, respectively. The absorption peaks at 1510 and 1350 cm^– 1^ are attributed to N–H and C–N bonds, respectively. The absorption peak at 820 and 1242 cm^– 1^ is originated from the epoxy group of epoxy resin. The absorption peak at 1460 cm^– 1^ is due to Methylene C-H bend vibration. There are two extra distinctive bands appear around 1737 and 2366 cm^− 1^ in the spectrum of Gr/Epoxy coating composites loaded with 0.04 wt%. These peaks are related to nitrogen functional groups which belong to the N-doped graphene; NH bending vibration of primary amine at 1783 cm^− 1^ and nitrile C≡N and/or isocyanate -N = C = O at 2340 cm^− 1^, as shown in the FTIR spectrum of Gr in Fig. 10b. Regarding the Gr/Epoxy composites, most of the influenced bands in their positions, as seen in the zoom in of the 1650–2400 cm^− 1^ FTIR regions in Fig. 10c. As the Gr loading in the composites coating increases, there is a red shift in the band related to the isocyanide/nitrile from 2340 up to 2357 cm^− 1^ and become more shifted to 2366 cm^− 1^, in the spectrum of Gr/Epoxy coating composites loaded with 0.02 and 0.04 wt%, respectively. Moreover, there is a blue shift in the band belong the NH bend of primary amine from 1783 downward to 1740 and 1737 cm^− 1^, in the spectra of Gr/Epoxy coating composites loaded with 0.02 and 0.04 wt%, respectively. This as compared with the spectrum of Gr in Fig. 10b. This most credibly due the hydrogen bonding formation between those N polar groups which belong to the functionalized graphen and the epoxy groups in the neat epoxy chains^35^. The farther the band is shifted, the more dense hydrogen bonds between the Gr and Epoxy are frormed. Thus, Gr/Epoxy coating composites loaded with 0.04 wt%, possess the densest filler-matrix hydrogen bonds a little bit higher than that which loaded with 0.02 wt%, as evidenced from bands positions shift in Fig. 10c. Interestingly, the band at 2,366 cm^− 1^ is increased in intensity as the Gr loading increases up to 0.04 wt%, which is indicative of enhanced nitrile/isocyanate bonding between the Gr network and the Epoxy, however, it seems such band intensity is comparable to that of neat epoxy, indicating no formation of hydrogen bonding between Gr and Epoxy and therefore weaker interfacial bonding between the two phases in Gr/Epoxy coating composites loaded with 0.01 and 0.03 Wt%. This could give a possible explanation of why the two Gr/Epoxy coating composites samples record the optimal values of the mechanical parameters as compared with the other filler content and with the neat epoxy as well.Whereas, Gr/Epoxy coating composites loaded with 0.03 wt% has unanimous behavior for all tests this could attribute to uncontrollable or unfavorable agglomeration of Gr within the Epoxy matrix during the curing process. This is intern reduce the Gr/ Epoxy interfacial interactions.

The incorporation of nitrogen-doped graphene (Gr) into epoxy coatings markedly improves both corrosion resistance and mechanical performance through a synergistic mechanism stemming from Gr’s distinctive structural and interfacial properties. Its two-dimensional planar morphology, high aspect ratio, and inherent impermeability contribute to the formation of tortuous diffusion pathways within the epoxy matrix, significantly impeding the ingress of corrosive agents such as water, oxygen, and chloride ions. This enhanced barrier effect is supported by improved hydrophobicity, as evidenced by contact angle measurements. Notably, the formulation containing 0.02 wt% Gr exhibited the highest hydrophobicity, with a 50% increase in contact angle (an increase of 31.04°) compared to the neat epoxy, indicating reduced surface wettability and improved water repellence. The optimized spatial distribution and dispersion of Gr further reduce permeation channels, while strong interfacial adhesion between Gr sheets and the epoxy matrix minimizes microvoid formation and delamination, contributing to enhanced coating integrity and durability.

From a mechanical standpoint, the uniform dispersion of Gr at ultra-low concentrations (< 0.04 wt%) facilitates efficient stress transfer across the filler-matrix interface, resulting in significant improvements in tensile strength, elongation, and toughness. Nitrogen functionalities on the Gr surface enhance interfacial bonding through hydrogen bonding and π–π interactions, which promote energy dissipation and reinforce the matrix under load. Gr also acts as a crack-arresting agent, dissipating mechanical energy and preventing crack propagation, thereby increasing the coating’s mechanical resilience and indirectly extending its corrosion resistance by delaying coating failure.

Additionally, Gr-reinforced coatings demonstrated superior flexibility and fracture toughness, characterized by increased elongation at break and higher energy absorption. These enhancements are attributed to the homogeneous distribution of Gr within the matrix, which ensures even stress distribution. The wrinkled morphology of Gr provides a larger interfacial area and improves filler-matrix adhesion, preventing filler pull-out under stress. Although minor reductions in modulus and tensile strength were observed in some Gr-loaded samples likely due to the bending and waviness of larger graphene sheets the 0.02 wt% Gr formulation exhibited optimal mechanical reinforcement, balancing strength and ductility effectively.

In finale, Gr functions as a multifunctional nanofiller, enhancing both barrier and mechanical properties of epoxy coatings. This dual functionality highlights its potential in the development of high-performance, environmentally friendly coatings for structural and marine applications. Gr’s ability to simultaneously improve corrosion protection and mechanical robustness positions it as a promising candidate for next-generation coating technologies.

**Table 4:** Corrosion and mechanical performance comparison of gr/epoxy coating with recent Graphene-Based and sustainable coatings.

Nanocomposites Coating	Method of preparation	Filler content (wt%)	Elongation at break (%)	Tensile Strength (MPa)	Ultimate elongation (%)	Contact angle	Ultimate toughness	Toughness at fracture (%)	Wear Index reduction (%)	Impact (Joule)	Bend test (mm)	Reference
Gr/Epoxy	One pot solvothermal treatment of chitozan for 4 h, at 270 °C	0.04 0.02	191± 6.3 158	17.7±3.6	603±5.3	93.05°±0.27°	431 573	755 993	70	18	< 5	Current study
(lignin-OH/graphene/ waterborne epoxy resin)	-lignin-OH was dissolveed in 40 mL of DMF and 50 g of 48% aqueous hydrogen bromide were added into flask to react at 115 °C for 20 h. -graphene from pristine graphite by exfoliation in water in ice bath sonicated for 6 h.	0.5	107.4 ± 5.8	48.91 ± 2.4		59.6°						^[36]^
Functional graphene oxide/epoxy nanocomposite	FGO was prepared according to an improved Hummers’ method	0.5 wt% GO FGO				69.5◦ 87◦						^[37]^
RGO/ waterborne soy oil epoxy (WSOE)		1.5 wt%								16.9	< 5	^[38]^
G/Epoxy	G was synthesized from residual agricultural biomass through pyrolysis) via mechanical and liquid phase exfoliation	0.1 wt%	77.2%	16.19	77.2	70.1		90.5				^[39]^
N-doped-GO@Zn nano-layers/epoxy composite	GO was prepared according to modified Hummer’s method, followed by hydrothermal treatment to obtain N-GO	0.2 wt%	61.5	46.48				309				^[12]^
GO-His/EP	GO was synthesized following a modified Hummer’s procedure, followed by Green reduction/functionalization of GO by histamine		1.76%	53.33 ± 2.85				35.11%				^[40]^
GNP/Epoxy	Purchased functionalized GNP with amine groups, (GNPNH_2_) thickness < 4 nm and an average lateral size of 1–2 μm	6 wt%	-40	48±1				-63.8				^[33]^

### Comparative analysis and discussion with recent studies

To benchmark the performance and sustainability of the nitrogen-doped graphene (Gr)/epoxy nanocomposite coating developed in this study, a comparative evaluation was conducted against recent graphene-based and eco-friendly coatings reported in the literature. A summary of the synthesis approaches, filler sources, loading levels, and key corrosion and mechanical performance indicators is provided in Table 4. The findings clearly demonstrate that the Gr/epoxy system presented herein offers exceptional multifunctional performance at ultra-low loading levels (0.02 wt%), which is significantly lower than those employed in previous studies (typically ≥ 0.5 wt%). This reduction in filler content not only preserves the workability of the epoxy matrix but also minimizes potential agglomeration issues that often compromise coating performance at higher loadings. From a mechanical perspective, the current formulation exhibited a substantial enhancement in ultimate toughness and elongation at break, far exceeding those of coatings reinforced with graphene synthesized from graphite or lignin. Specifically, a 993% increase in toughness at fracture and a 573% increase in ultimate toughness were observed with only 0.02 wt% Gr. These superior results are attributed to the homogeneous dispersion of Gr nanosheets, their large lateral dimensions, and wrinkled morphology, which together contribute to better stress transfer, crack bridging, and energy dissipation across the matrix–filler interface.

In terms of corrosion protection, the coating displayed a significant reduction in wear index (up to 70% lower than neat epoxy) and enhanced hydrophobicity, as evidenced by a 50% increase in contact angle. These effects are ascribed to the tortuous diffusion paths formed by the Gr layers, which effectively impede the ingress of corrosive species, and to the high nitrogen doping level (8.69 at%), which enhances interfacial bonding and barrier effects. Notably, this level of nitrogen incorporation is much higher than what has been reported in other studies using similar green precursors (typically 1–3 at%), and is achieved through a solvent-free, one-pot solvothermal synthesis using chitosan—a low-cost, renewable biopolymer.

Moreover, unlike traditional methods that often rely on toxic oxidizing agents (e.g., KMnO₄, HNO₃) or multi-step treatments, the current Gr synthesis approach is simple, green, and scalable, requiring no post-processing or hazardous chemicals. This aligns with the global push toward sustainable materials development and low-impact manufacturing for high-performance coatings.

Overall, the comparison highlights that the chitosan-derived Gr coating not only matches but outperforms existing graphene-based coatings in both mechanical and corrosion resistance properties, and does so using an eco-friendly synthesis route with significantly reduced material usage. These findings support the potential of this material system for advanced protective applications in marine, structural, and aerospace environments, where both durability and sustainability are critical.

**Highlighting novelty, efficiency, and environmental benefits.**

Compared to recent sustainable coatings, our epoxy nanocomposite reinforced with chitosan-derived N-doped graphene provides a unique combination of mechanical robustness and corrosion resistance using a low nanofiller load (< 0.1 wt%). Unlike ceramic-heavy or additive manufacturing-based systems 6, 42, 45, our coating 41maintains flexibility and processability. Smart or hybrid coatings 43, 44 offer promising multifunctionality but often rely on complex synthesis routes or expensive components. In contrast, our green, single-step solvothermal synthesis offers scalability and cost-effectiveness, marking a significant advancement in the design of sustainable protective coatings. A summary for the segregation of recent sustainable coating studies to emphasize the novelty of our study is summarized in Table 5.

**Table 5:** The segregation of recent sustainable coating studies to emphasize the novelty of our study:

Coating System	Sustainability Focus	Limitations	Comparison & Novelty	Reference
SPD-treated Mg alloy passivation	Low-energy mechanical processing + green processing (no chemicals used)	Limited mechanical reinforcement; metal-only system	While their passive layer improves corrosion, ours adds *epoxy–graphene* barrier functionality, outperforming in both corrosion and mechanical tests. Our chitosan-derived N-doped graphene/epoxy system offers better barrier properties and integrates bio-based nanofillers for corrosion protection.	^[41]^
NiCrCr₃C₂hBN coatings	Wear resistance with ceramic fillers Thermal stability and wear resistance	Inorganic-heavy; less sustainable	Our graphene-based coating achieves superior corrosion resistance with lower filler load and is more environmentally benign, improved corrosion inhibition, and avoids heavy metals or ceramics.	^[42]^
Ceramic coatings in subcritical water(in harsh environments)	Supercritical aqueous corrosion (Resistance to supercritical conditions)	Brittleness, energy-intensive processing	While effective in niche environments, ceramic layers are brittle.Our coating remains effective under standard corrosive conditions with flexibility, suitable for real-world applications without special processing.	^[6]^
Datadriven R&D for functional coatings (Smart stimuli-responsive nanocoating)	Digital materials discovery Switchable hydrophobicity; environmental stimuli responsiveness	Complex synthesis; potential cost issues	Their review highlights the platform; our work offers a validated, experimentally optimized *green graphene–epoxy composite*, illustrating data-informed design principles we plan to adopt.Our system uses a single-step solvothermal synthesis with renewable precursors, offering a more scalable, eco-friendly route with mechanical and anticorrosive synergy.	^[44]^
rGO/gC₃N₄/Ag for water treatment	Remediation Photocatalytic/antimicrobial coatings	Multi-component, higher cost	Focused on catalysis, this study uses higher graphene loads; our coating retains environmental and mechanical performance with minimal graphene (< 0.1 wt%), reducing material use and cost.	^[45]^
AM Al–Si alloys Additive manufactured Al–Si alloys	Microstructuremech. Behavior Green AM processing	Material/system-specific; high energy input	Their insights illuminate structure–property relationships; we extend this by showing that graphene reinforcement enhances epoxy coating strength and toughness, a novel materials-design approach, Our approach uses bio-derived nanofillers in a conventional coating matrix, applicable to a wider range of surfaces with simpler processing.	^[43]^
Energetic coating using Al@AP with GO-based energetic coordination polymer (ECP)	Focus on improving combustion energy, not corrosion protection; involves metal-based energetic salts	Requires multiple synthesis steps; involves energetic and potentially hazardous materials	Designed for high-temperature energetic applications, while our study Targets corrosion protection and mechanical reinforcement - Significant toughness and wear improvements at ultra-low filler content - Ambient-condition performance - Eco-friendly and safe for large-scale use	^[46]^

## Conclusions

This study demonstrates the successful development of an environmentally sustainable epoxy nanocomposite coating reinforced with nitrogen-doped graphene (Gr) synthesized through a simple, green, and cost-effective solvothermal method using chitosan as a carbon and nitrogen source. The structural and morphological characterization confirmed the formation of well-defined graphene nanosheets with extended lateral dimensions and folded sheet morphology. When incorporated into epoxy matrices at ultralow concentrations (< 0.04 wt%), the N-doped graphene imparted remarkable improvements in both anticorrosive and mechanical performance. The nanocomposite coatings exhibited significant enhancements in corrosion resistance, as demonstrated by salt spray, adhesion, abrasion, and impact resistance tests. Moreover, the inclusion of only 0.02 wt% Gr led to a dramatic increase in mechanical properties, particularly ultimate toughness and toughness at fracture, which enhanced by 573% and 993%, respectively, compared to the neat epoxy. These enhancements are attributed to strong Gr/epoxy interfacial bonding and the tortuous crack propagation path enabled by the fine dispersion of the nanofiller. The outcomes of this work clearly establish the potential of chitosan-derived N-doped graphene as a multifunctional reinforcement for epoxy coatings, offering both environmental sustainability and superior structural performance. This work provides a promising framework for the development of green, multifunctional composite materials suitable for advanced protective and structural applications.

## Electronic supplementary material

Below is the link to the electronic supplementary material.

Supplementary Material 1

## Data Availability

Our manuscript has data included as supplementary materials.

## Declarations

### Future perspective and recommendation

Owing to high aspect ratio of Gr which leads to agglomeration in staked form. Besides, the dispersion of Gr is more difficult in a viscous medium, due to the sharp increase of the polymer viscosity with increasing the nanofiller loading percentage. Hence, Accordingly, here it was a limitation for using very low Gr concentrations don’t exceed 0.04 wt%. Thus, we recommend employing the sonication parameters carefully selected, including the subjected power output of probe sonicator to o enhance the interactions between Gr and Epoxy and improve the distribution and dispersion of Gr within the epoxy resin. Future work should investigate the long-term performance under simulated environmental conditions such as wet-dry cycles, microbial exposure, or thermal aging to better understand performance limits and potential mitigation strategies.
