# Correction: Environmentally sustainable epoxy nanocomposite coating reinforced with chitosan derived nitrogen doped graphene for enhanced corrosion resistance and mechanical performance

**DOI:** 10.1038/s41598-025-22456-7

**Published:** 2025-10-07

**Authors:** Marwa Adel, Dalia S. Fathy, Osama Abo El-eneen

**Affiliations:** 1 Fabrication Technology Department, Advanced Technology and New Materials Research Institute, City of Scientific Research and Technological Applications (SRTA City), New Borg El-Arab, Alexandria, 21934 Egypt; 2 Petroleum Applications Department, Egyptian Petroleum Research Institute (EPRI), Nasr City, Cairo, 11727 Egypt; 3 Petrochemicals Department, Polymer Laboratory, Egyptian Petroleum Research Institute (EPRI), Nasr City, Egypt

Correction to: *Scientific Reports* 10.1038/s41598-025-11204-6, published online 20 August 2025

The original version of this Article contained errors in Equations 1 and 2, where

Equation 1 was incorrectly given as:

$$\:Mass\:loss={M}_{intact\:coating}+\:{M}_{damaged\:coating}$$

Equation 1 now reads:

$$\:Mass\:loss={M}_{intact\:coating}-\:{M}_{damaged\:coating}$$

Equation 2 was incorrectly given as:

$$\:WI=\frac{{M}_{intact\:coating}+\:{M}_{damaged\:coating}}{Number\:of\:cycles\:of\:abrasion}$$

Equation 2 now reads:

$$\:WI=\frac{{M}_{intact\:coating}-\:{M}_{damaged\:coating}}{Number\:of\:cycles\:of\:abrasion}$$

The original Article has been corrected.

