# Supplementary material for: Environmentally sustainable epoxy nanocomposite coating reinforced with chitosan derived nitrogen doped graphene for enhanced corrosion resistance and mechanical performance
Source: Sci Rep. 2025 Aug 20;15:30617. doi: 10.1038/s41598-025-11204-6 (PMC12368276; doi:10.1038/s41598-025-11204-6)
Supplement: Supplementary file 1 — Supplementary Material 1 [file 41598_2025_11204_MOESM1_ESM.doc]

**Supplymentary Materials (S)**

**Environmentally sustainable epoxy nanocomposite coating reinforced with chitosan-derived N-doped graphene for superior corrosion resistance and mechanical enhancement**

Marwa Adel^a,b^*, Dalia S. Fathy^c^ and Osama Abo El-eneen^b^

**
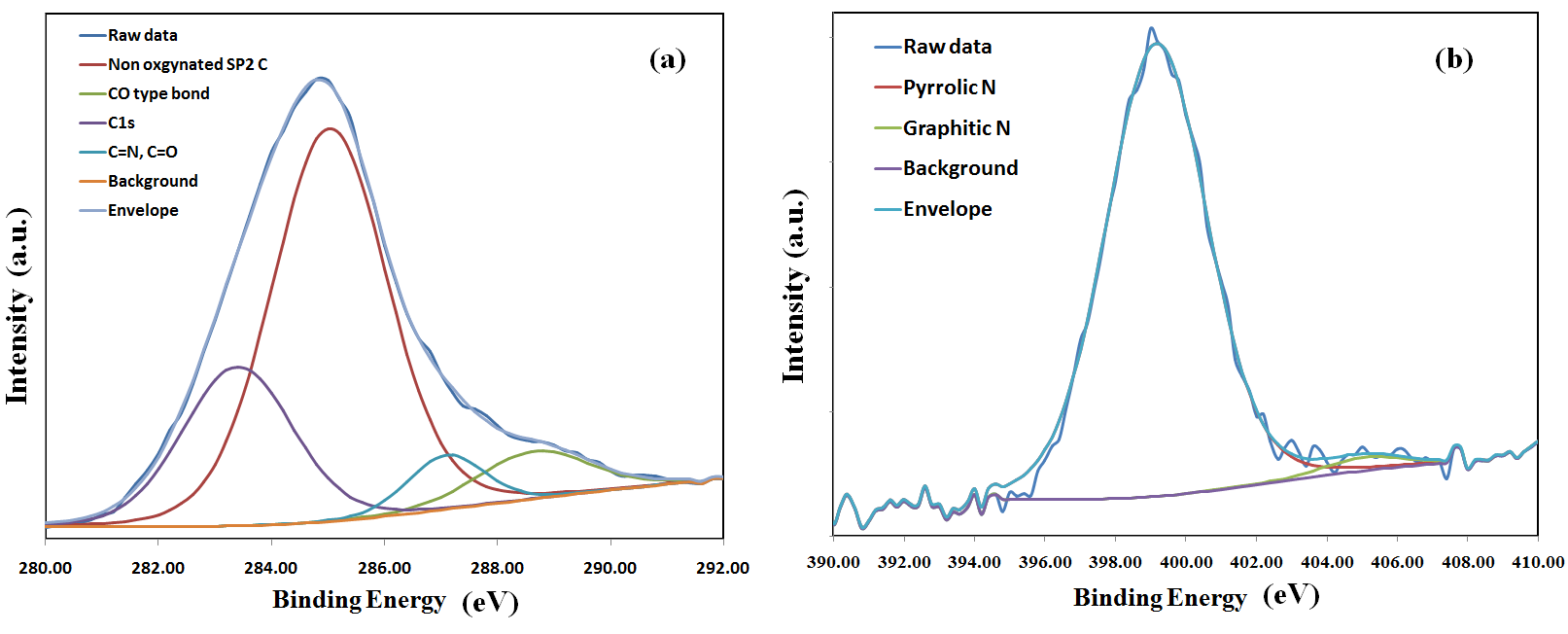
**

***S1*. XPS spectra of NG-0.3-270 (a) C1s and (b) N1s.**


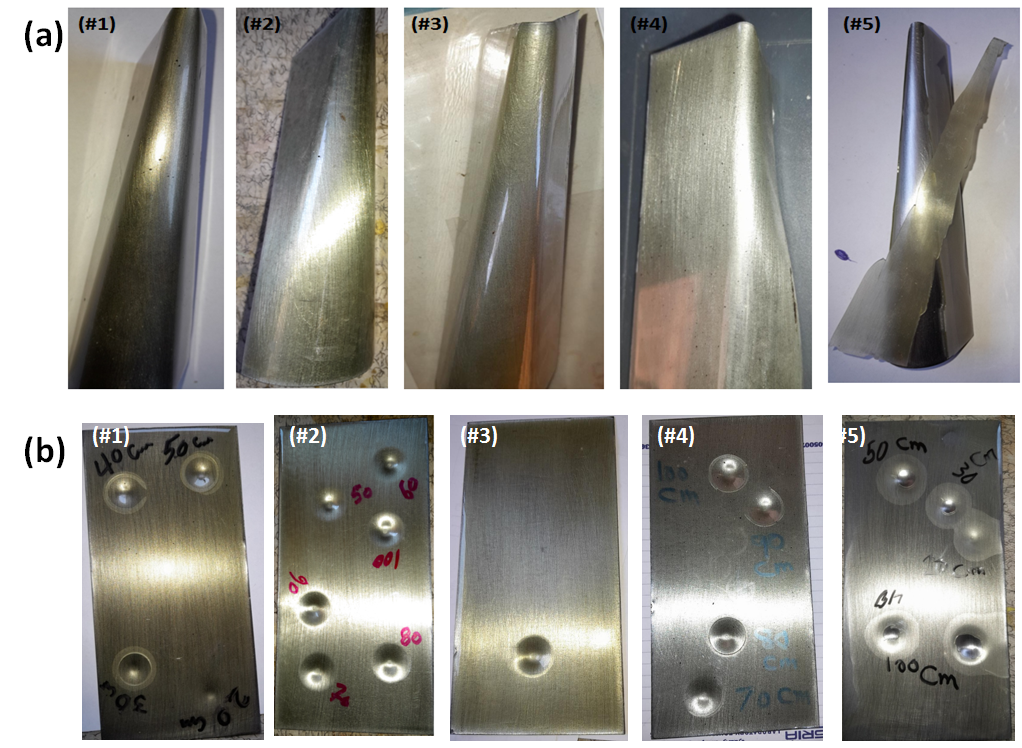


***S2.*** Macrophotographs of samples #1–5 *of (a) Bending and (b) impact resistance test results*


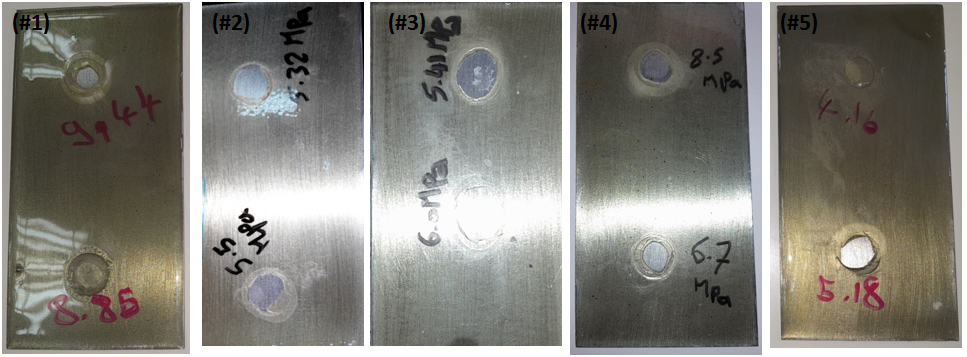


***S3. The adhesion of the painted samples tested by pull-off test***

***Table S1. The water contact angle pictures of the neat epoxy and Gr/Epoxy coating samples with their standard deviations gotten from 10 various measurements.***

| **Coating** | **Contact Angle (degree)** |
| --- | --- |
| Neat Epoxy | 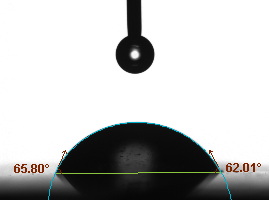 62.01 °±1.16° |
| Gr/Epoxy (0.01%) | 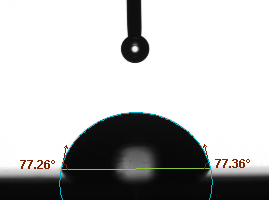 77.36 °±.0.43° |
| Gr/Epoxy (0.02%) | 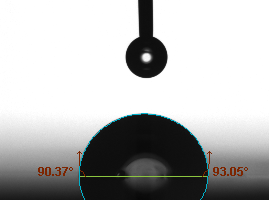 93.05°±0.27° |
| Gr/Epoxy (0.03%) | 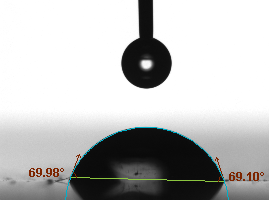 69.10°±1.66° |
| Gr/Epoxy (0.04%) | 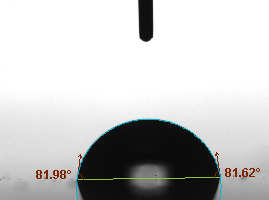 81.62°±0.31° |
